# Quorum-sensing- and type VI secretion-mediated spatiotemporal cell death drives genetic diversity in *Vibrio cholera*

**DOI:** 10.1016/j.cell.2022.09.003

**Published:** 2022-09-26

**Authors:** Ameya A. Mashruwala, Boyang Qin, Bonnie L. Bassler

**Affiliations:** 1 Department of Molecular Biology, Princeton University, Princeton, NJ 08544, USA; 2 Department of Mechanical and Aerospace Engineering, Princeton University, Princeton, NJ 08544, USA; 3 The Howard Hughes Medical Institute, Chevy Chase, MD 20815, USA; 4 Lead Contact

## Abstract

Bacterial colonies composed of genetically identical individuals can diversify to yield variant cells with distinct genotypes. Variant outgrowth manifests as sectors. Here, we show that Type VI secretion system (T6SS)-driven cell death in *Vibrio cholerae* colonies imposes a selective pressure for the emergence of variant strains that can evade T6SS-mediated killing. T6SS-mediated cell death occurs in two distinct spatiotemporal phases, and each phase is driven by a particular T6SS toxin. The first phase is regulated by quorum sensing and drives sectoring. The second phase does not require the T6SS-injection machinery. Variant *V. cholerae* strains isolated from colony sectors encode mutated quorum-sensing components that confer growth advantages by suppressing T6SS-killing activity while simultaneously boosting T6SS-killing defenses. Our findings show that the T6SS can eliminate sibling cells, suggesting a role in intra-specific antagonism. We propose that quorum-sensing-controlled T6SS-driven killing promotes *V. cholerae* genetic diversity, including in natural habitats and during disease.

## In brief

Type VI secretion system-mediated cell death in *Vibrio cholerae* colonies occurs in two space- and time-differentiated phases that cause death along the colony rim and interior, respectively. Cell death at the colony rim is a consequence of kin-killing and is proposed to drive genetic diversity in nature and during disease.

## Graphical abstract

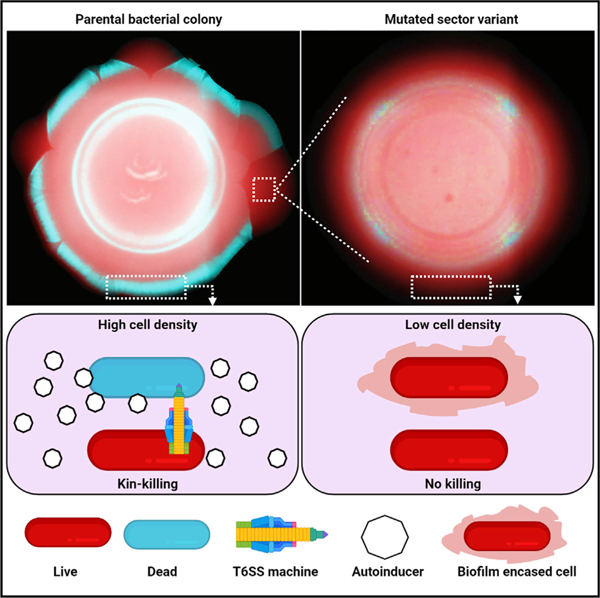

## INTRODUCTION

**Figure 1.**
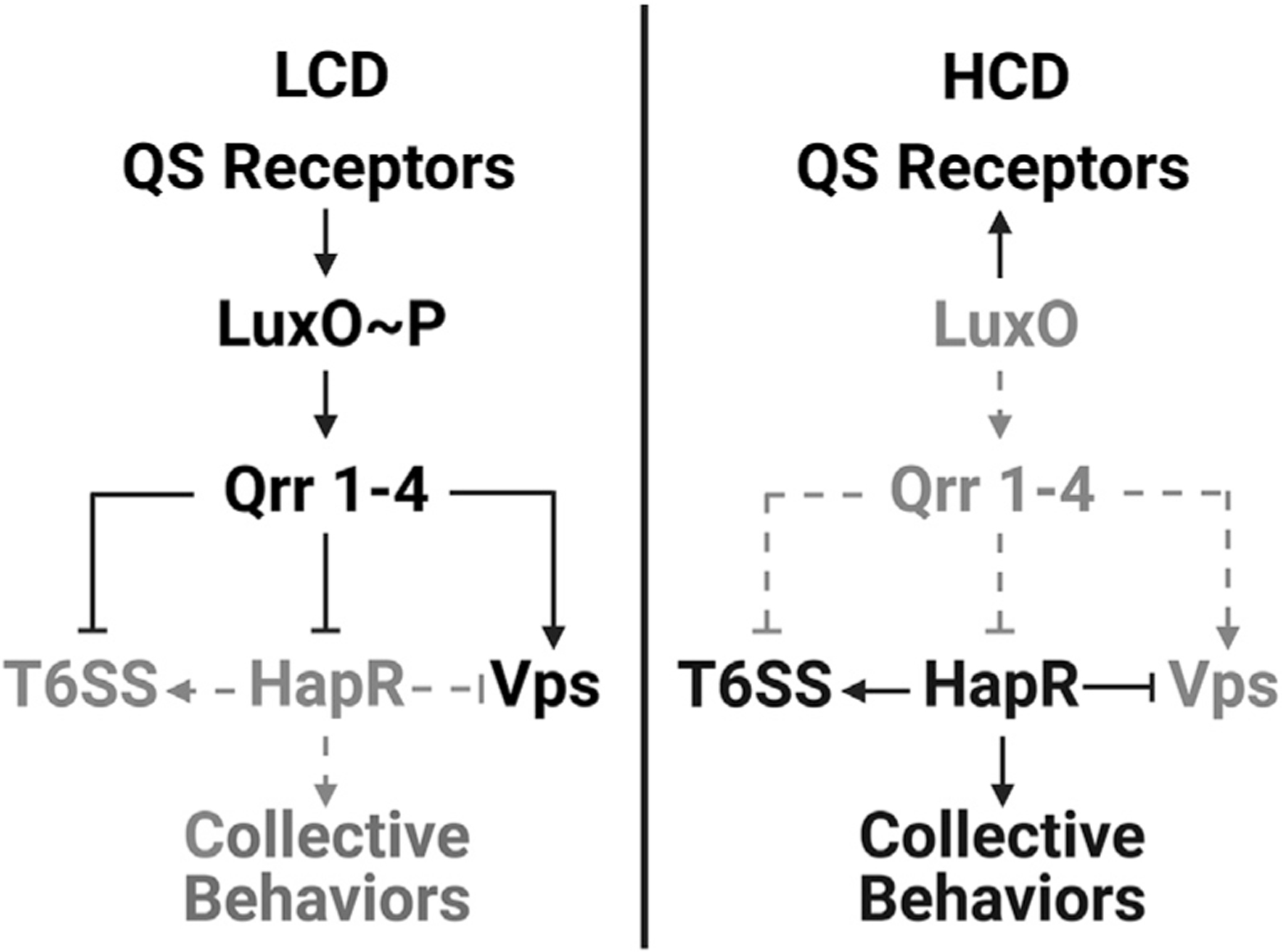
Simplified scheme for *V. cholerae* QS regulation of *t6ss* and *vps* genes See text for details. See also Figure S1.

Bacteria track cell population density using a process called quorum sensing (QS). QS relies on the production, release, accumulation, and detection of extracellular signal molecules called autoinducers (AIs). QS enables groups of bacteria to synchronize gene expression and collectively enact processes that demand many cells working together to make the task successful (Papenfort and Bassler, 2016; Waters and Bassler, 2005). In *Vibrio cholerae*, the causative agent of the cholera disease and the model bacterium used for the present work, two parallel QS pathways funnel information contained in AIs to a shared transcription factor called LuxO (Figure 1) (Miller et al., 2002). At low cell density (LCD), in the absence of AIs, the AI receptors act as kinases ferrying phosphate to LuxO (Wei et al., 2012). LuxO~P activates transcription of genes encoding four small RNAs (sRNA) called Qrr1–4. Qrr1–4 repress translation of HapR, encoding the master high cell density (HCD) QS regulator (Lenz et al., 2004). At HCD, when AIs have accumulated, the receptors act as phosphatases (Neiditch et al., 2005). LuxO is dephosphorylated and inactive. Production of the Qrr sRNAs is halted, HapR is translated, and it promotes expression of QS-controlled genes specifying collective behaviors (Lenz et al., 2004).

The *V. cholerae* type VI secretion system (T6SS) is a QS-regulated, contact-dependent protein delivery system that enables attack and elimination of competitor cells (MacIntyre et al., 2010; Pukatzki et al., 2006; Shao and Bassler, 2014). Briefly, T6SS structural components are assembled into a membrane-spanning spear-like device loaded with toxic effector proteins (Ho et al., 2014; Russell et al., 2014; Wang et al., 2019). The apparatus shoots the effectors into competitor cells by puncturing their cell walls. To prevent self-killing, T6SS-active bacteria produce immunity proteins that inactivate the toxic effector proteins (Ho et al., 2014; Russell et al., 2014; Wang et al., 2019). Other defenses, such as production of exopolysaccharide or capsular polysaccharide also protect against incoming T6SS attacks (Flaugnatti et al., 2021; Hersch et al., 2020; Hood et al., 2010; Toska et al., 2018). In *V. cholerae*, *t6ss* genes are located in one large and three auxiliary clusters (Figure S1) (Metzger et al., 2016). The T6SS machinery is largely conserved among *V. cholerae* strains, however, its expression and regulation are strain specific. Important for this work is that a *cis*-acting single nucleotide polymorphism (SNP) causes the El Tor environmental isolate 2740–80 to express its *t6ss* genes (Dörr et al., 2022; Ng et al., 2022). By contrast, the closely related pandemic isolate C6706 that lacks the *cis*-acting SNP does not express *t6ss* genes under laboratory conditions. In *V. cholerae* strains that do express *t6ss* genes, at LCD, the Qrr sRNAs repress *t6ss* expression by two mechanisms: they directly repress the large *t6ss* cluster and they function indirectly by repressing *hapR*, an HCD activator of the auxiliary *t6ss* gene clusters (Shao and Bassler, 2014). Direct cell-to-cell contact is required for T6SS toxin deployment (MacIntyre et al., 2010). Thus, restricting maximal production of the T6SS machinery to HCD could boost killing efficiency and be bioenergetically favorable.

Bacterial colonies are structured communities in which cells occupy micro-habitats with varying physical and chemical compositions (Bjedov et al., 2003; Hashuel and Ben-Yehuda, 2019; Saint-Ruf et al., 2014). These heterogeneous environments impose distinct selective pressures for advantageous variant genotypes to arise. Often, the appearance of variants manifests as colony sectors. In *Staphylococcus aureus* and *V. cholerae*, such variants display increased virulence in animal models and/or resistance to antibiotics (Finkelstein et al., 1992; Holmes et al., 1975; Koch et al., 2014; Servin-Massieu, 1961). Understanding the mechanisms driving colony variation could provide insight into the general emergence of new genotypes and their corresponding traits.

Here, we investigate the molecular mechanisms underlying colony sectoring in *V. cholerae*. We find that sectoring in colonies initially composed of genetically identical cells is preceded by T6SS-mediated cell death that occurs in two different spatiotemporal phases, each driven by distinct T6SS effectors. Tracking cell death using fluorescence microscopy shows the first phase occurs along the colony rim. During this cell death phase, QS, *t6ss*, and *vps* genes exhibit regional differences in expression, which sets the cell death spatial pattern. T6SS-driven killing imposes a selective pressure for variant strains containing QS-inactivating mutations to arise. Loss of QS activity confers protection from T6SS-killing by two mechanisms. First, production of vibrio polysaccharide (Vps), normally a QS-repressed trait, increases and Vps acts to shield cells from incoming T6SS attacks. Second, elimination of QS-dependent activation of *t6ss* gene expression reduces overall T6SS-killing events. These changes confer regional growth advantages to the QS loss-of-function mutants that manifest as outgrowth into colony sectors. While requiring a T6SS toxin, the second cell death phase—which occurs in the colony interior—does not affect sectoring, and, intriguingly, does not rely on the T6SS-injection apparatus. We propose that T6SS-driven intra-specific antagonism promotes *V. cholerae* genetic diversity, including in natural habitats and during disease, both of which are well known to select for *V. cholerae* variants that display reduced QS activity.

## RESULTS

### *Vibrio cholerae* undergoes QS-dependent sectoring

Certain Vibrionaceae bacteria, including strains of *Vibrio vulnificus*, *Vibrio parahaemolyticus*, *Vibrio harveyi*, and *V. cholerae*, form colonies that, over time, develop sectors that differ in opacity compared to the original colony (Chatzidaki-Livanis et al., 2006; Finkelstein et al., 1992; McCarter, 1998; Simon and Silverman, 1983). Presumably, other characteristics not visible to the eye are also regionally altered in the sectors and/or elsewhere in such colonies. The mechanism driving particular vibrios to form heterogeneous communities is not known. Here, we investigate the molecular underpinnings of colony sectoring using *V. cholerae* as our model system.

**Figure 2.**
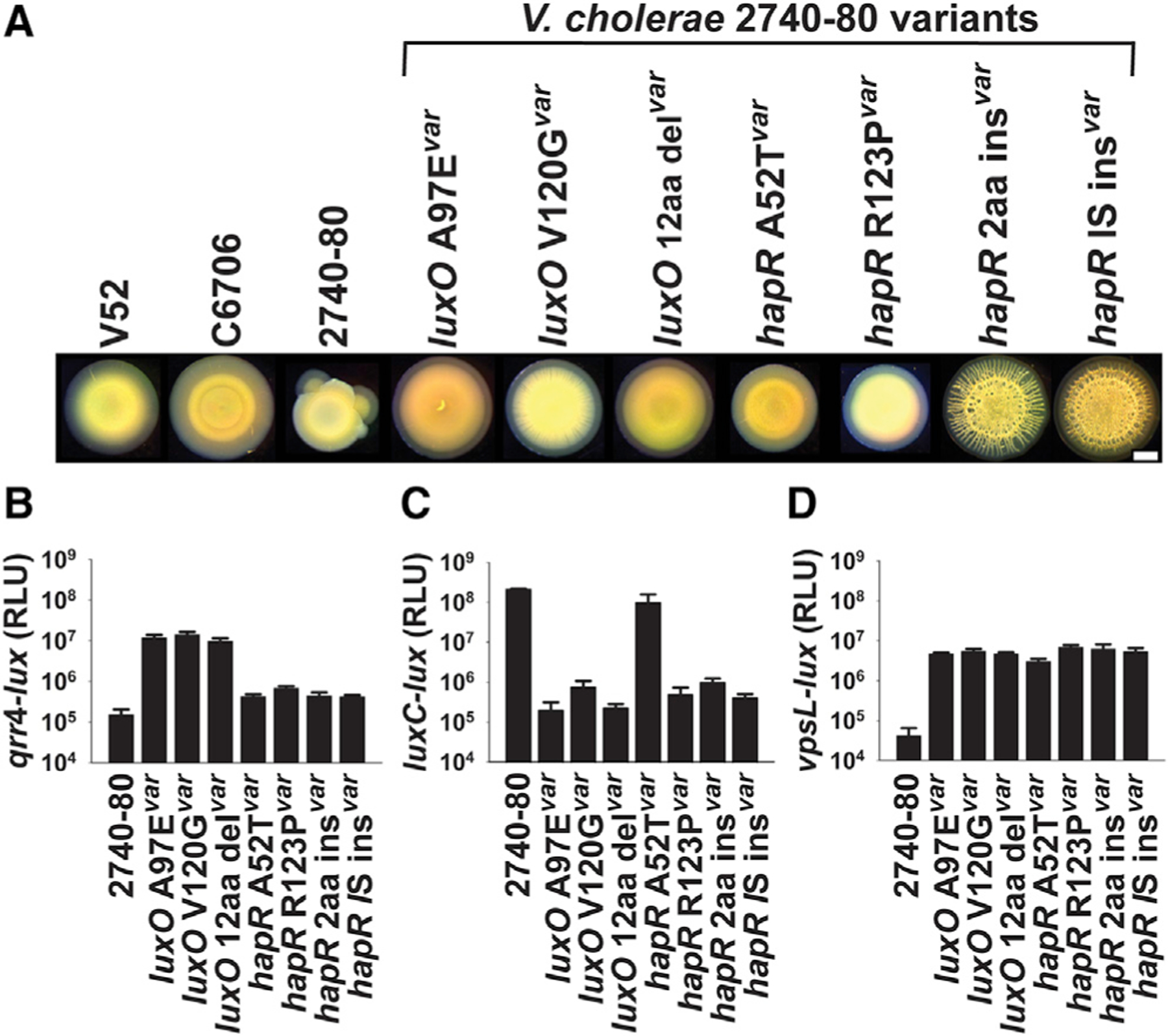
*V. cholerae* 2740–80 undergoes QS-dependent sectoring (A) Brightfield stereo-microscope images of 2-day-old colonies of the indicated *V. cholerae* strains. Scale bar = 1 mm. (B–D) Transcriptional activities of (B) *qrr*4-*lux*, (C) *luxC-lux*, and (D) *vpsL*-*lux* in the indicated strains. The ‘‘var’’ suffix denotes variant strains. In (B)–(D), data represent average values from biological replicates (n = 4), and error bars show SDs. See also Figure S2 and Table S1.

Following ~2 days of incubation on solid Luria-Bertani (LB) medium, colonies of the O37 serogroup strain V52 and *V. cholerae* El Tor biotype strain C6706 did not sector and their morphologies remained uniformly translucent (Figure 2A). By contrast, the closely related *V. cholerae* El Tor biotype strain 2740–80 formed opaque sectors that were distinct from the translucent morphology of the initially growing colony (Figure 2A). We purified isolates from ten individual *V. cholerae* 2740–80 sectors. Each isolate formed a homogeneous opaque colony that did not sector, suggesting that these variants have acquired mutations that lock them into the phenotype of the sector. The levels of opacity differed between isolates, with the most extreme variant colonies displaying wrinkled morphologies (Figure 2A shows seven of these isolates). In *V. cholerae*, over-production of Vps confers an opaque and wrinkled colony appearance, indicating that the variants from the sectors may have acquired mutations that drive increased Vps production. To investigate this possibility, we used whole genome and Sanger sequencing to pinpoint the mutations that had occurred in nine of the isolated *V. cholerae* 2740–80 variants. Seven variants possessed a single nucleotide change, a deletion, an insertion, or an insertion-element aided interruption in genes encoding the *V. cholerae* master QS regulators LuxO (3 variants) and HapR (4 variants) (Table S1 and designated in Figure 2A). One variant acquired a mutation in the 3′ UTR of the gene encoding the cold shock protein CspA, and the final variant had a mutation in *pyrG* encoding CTP synthase (Table S1). Here, we focus on how alterations in QS drive changes in *V. cholerae* colony morphology and sectoring capability. We remark on the *cspA* and *pyrG* mutants in the Discussion.

To explore the connection between QS, sectoring, and colony morphology, we assessed whether the mutations in *luxO* and *hapR* that arose in the sectors specified gain- or loss-of-function alleles. Both LuxO and HapR are transcription factors. To measure their activities, we engineered luciferase (*lux*) transcriptional reporter fusions to well-characterized LuxO-controlled (*qrr*4) and HapR-controlled (*luxC*) promoters (designated *qrr*4-*lux* and *luxC-lux*, respectively). First, regarding LuxO: LuxO is phosphorylated and activates *qrr*4 transcription at LCD (Figure 1). The *V. cholerae* 2740–80 variants harboring mutations in *luxO* expressed ~100-fold more *qrr*4-*lux* than did *V. cholerae* 2740–80 at HCD. This result shows that the *luxO* variants are gain-of-function alleles (Figure 2B). Regarding HapR: HapR is produced and functions at HCD, when it activates transcription of genes (Figure 1). All of the *hapR* variants except one expressed ~100–1,000-fold less *luxC-lux* than did *V. cholerae* 2740–80 at HCD, showing that the HapR variants are either attenuated- or loss-of-function alleles (Figure 2C). Indeed, the colony morphologies of the loss-of-function *hapR* variants mimicked that of a ∆*hapR* strain (see Figure S2A for examples). Only the HapR A52T variant did not display an altered *luxC-lux* level (Figure 2C). HapR A52T has been studied previously. The A52T alteration affects HapR binding to DNA to different extents at different target promoters, but it does not affect binding to the *luxC* promoter (van Kessel et al., 2013). Thus, all but one of the QS mutants identified from the colony sectors ‘‘lock’’ the cells into the LCD QS mode.

QS promotes Vps production at LCD in *V. cholerae* (Hammer and Bassler, 2003). To connect the QS locked-LCD variant phenotypes to their opaque/wrinkled morphologies, we introduced a *vpsL-lux* transcriptional fusion into the strains and measured the output. *vpsL* encodes a biosynthetic protein required for Vps production. All of the LCD-locked QS variants displayed increased *vpsL-lux* expression compared with *V. cholerae* 2740–80 (Figure 2D). Thus, the LCD-locked QS states of the variants increases Vps production, and excess Vps converts the colonies from translucent to opaque/wrinkled. Importantly, although the colony wrinkling morphologies of the LCD-locked QS variants differed one from the other, none of the variant colonies sectored (Figure 2A).

Collectively, the above results suggest that the HCD QS mode drives colony sectoring. Moreover, the sectors contain cells with genotypes that differ from that of the parent strain and the different mutations in the cells in the sectors underlie their distinct morphologies. Because the QS receptors funnel all sensory information to LuxO, and LuxO functions upstream of HapR in the cascade (Figure 1), in the remainder of this work, we focus on the *luxO* variant strains to understand how the LCD-locked state influences colony sectoring.

### T6SS-activity drives spatiotemporal cell death that precedes colony sectoring

*V. cholerae* 2740–80, which sectors, highly expresses the genes encoding its T6SS, whereas *V. cholerae* C6706, which is also an El Tor biotype strain but does not sector, does not express *t6ss* genes under laboratory conditions. *V. cholerae* uses Vps as a physical barrier to block T6SS attacks (Toska et al., 2018). In *cholerae* 2740–80, at LCD, LuxO~P activates *vps* gene expression and represses *t6ss* gene expression (Figure 1) (Hammer and Bassler, 2003; Shao and Bassler, 2014). Thus, in our *V. cholerae* 2740–80 LCD-locked LuxO QS variants, *vps* expression is higher (Figure 2D). Similarly, at HCD, HapR represses *vps* expression; so in our *hapR* loss-of-function mutants, *vp*s expression increases (Figure 2D). Based on these patterns, we wondered whether cells in colonies of *V. cholerae* 2740–80 undergo T6SS-dependent killing. If so, individual cells that acquire mutations, such as in QS components that confer an increased ability to produce Vps, would reap growth advantages because they could use Vps to evade T6SS killing. This growth advantage could manifest in outgrowth as a sector. We designed experiments to test these ideas.

**Figure 3.**
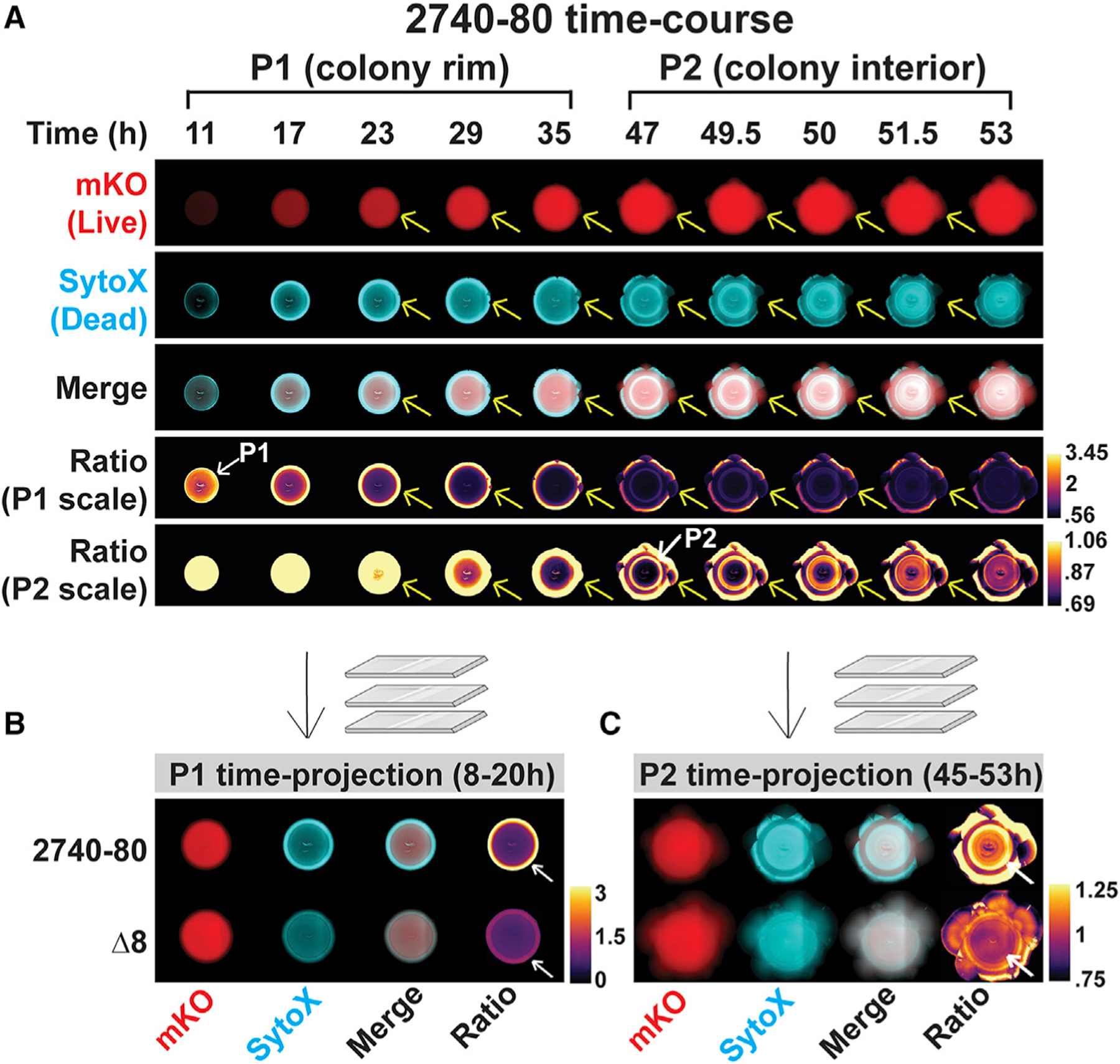
Two phases of spatiotemporal cell death occur in *V. cholerae* 2740–80 and Phase 1 precedes colony sectoring (A) Images from selected time points during growth of *V. cholerae* 2740–80 constitutively producing mKO, which marks live cells. Dead cells are marked with SytoX stain. Yellow arrows follow one sector as it emerges and expands. Two sets of ratio images are presented to aid visualization of cell death during the Phase 1 (denoted P1 scale) and Phase 2 (denoted P2 scale) time periods. The white arrow with the P1 designation that points to the colony rim highlights the region where maximal Phase 1 cell death occurs. The white arrow with the P2 designation that points to the ring in the colony interior shows the Phase 2 cell death region. In addition to showing each phase of cell death, the P1 scale best depicts that the colony rim is enriched in dead cells while the P2 scale best depicts that the sectors contain primarily live cells. (B and C) Time-projections for Phase 1 (B) and Phase 2 (C) cell death for the indicated strains. White arrows as in (A). See also Figure S3 and Video S1.

**Figure 4.**
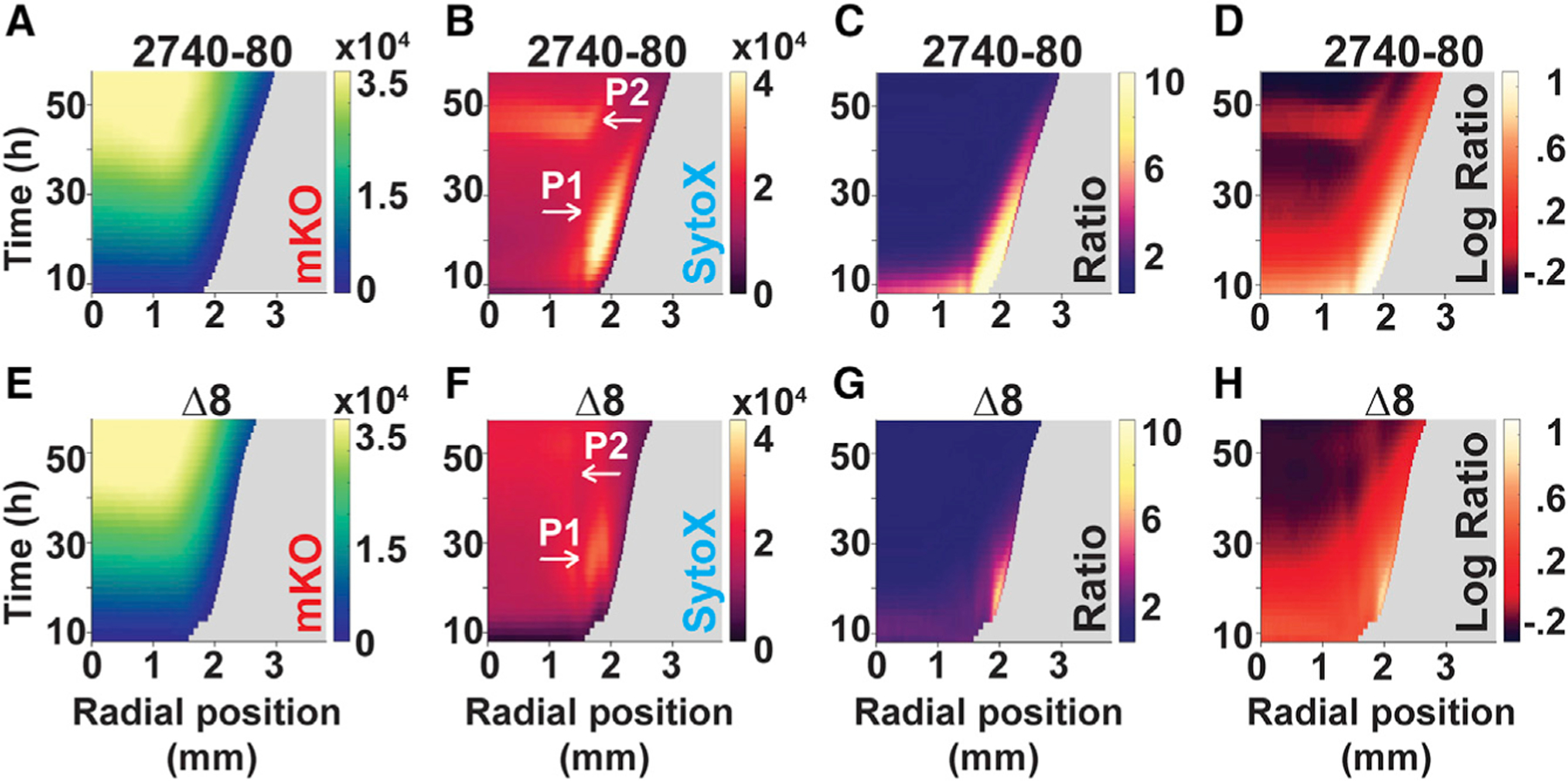
T6SS activity drives each cell death phase in *V. cholerae* 2740–80 (A–H) Space-time kymographs from the indicated channels and strains taken from regions lacking sectors. Kymographs in (C) and (G) display linear ratio data for visualization of Phase 1 cell death. Logarithmic ratio data are presented in (D) and (H) for emphasis of Phase 2 cell death. The X axis on each kymograph indicates the radial position in the colony at which intensity was quantified. The center of the colony is at 0 mm and the colony rim is at ~3 mm. Phase 1 cell death is indicated with the white arrows labeled P1 in (B) and (F), and is also visible in panels (C), (D), (G), and (H) along the colony rim as the yellow colored regions. Phase 2 cell death is indicated with the white arrows labeled P2 in (B) and (F) and is also visible in (D) as the red colored region in the colony interior. In all panels, intensities or ratios are color-mapped and the scale bars represent color:intensity. Intensity ratios were obtained by dividing the intensities from the dead-cell channel by that from the corresponding live-cell channel. Kymographs from one colony are representative of results from 3 to 9 colonies for each strain. See also Figure S3 and Video S1.

First, we assessed whether colonies of *V. cholerae* 2740–80 undergo T6SS-dependent killing and, if so, whether this affects colony sectoring. To do this, we quantified cell death in colonies of *V. cholerae* 2740–80 and in an isogenic strain lacking all four pairs of T6SS effector-immunity proteins (hereafter: ∆8 strain). We used time-lapse fluorescence microscopy to track live and dead cells and colony sectoring. For this analysis, all cells constitutively produced the mKO fluorescent protein (Red) to enable imaging of live cells and we used the fluorescent dye SytoX (Cyan) to mark dead cells. To enable visualization and fluorescence quantitation across the colonies, including in regions with sectors, we collapsed the time series data into single images by generating projections across time. Representative time series images are displayed in Figure 3A, while Figures 3B and 3C show the time projections. To quantify spatiotemporal cell death in non-sectored regions, we reduced the time-lapse data into space-time kymographs (Figure 4). In both the time-projections and the kymographs, data were mapped using colors as quantitative readouts for intensities. There are many features in the images that differ between the strains under study. We focus on only four of those features here: region-dependent cell death, time-dependent cell death, T6SS-mediated cell death, and sectoring.

We first discuss the results from *V. cholerae* 2740–80. The colonies displayed two phases of cell death, which we call ‘‘Phase 1’’ and ‘‘Phase 2’’, visible in the time projections and kymographs as regions exhibiting increased SytoX-dependent fluorescence relative to mKO fluorescence. Cell death during Phase 1 occurred between ~8 and 40 h and was concentrated predominantly along the periphery of the colony (first four rows in Figure 3A, top row Figure 3B, and Figures 4A–4C; indicated with the white arrows and the designation P1; Figures S3A–S3E and Video S1). At ~44 h, Phase 2 of cell death initiated in the colony interior as a ring and propagated in both the inward and outward directions in an apparent wave-like manner (first three and fifth rows in Figure 3A, top row Figure 3C, and Figures 4A, 4B, and 4D; indicated with the white arrows and the designation P2; Figures S3A–S3E, and Video S1). Importantly, ratio-metric kymograph and time-projection analyses of the dead and live cell distributions in the colony (SytoX/mKO) confirmed that the cell death patterns in the different regions and at the different times are not due to differences in cell numbers but, rather, are a consequence of alterations in the ratios of live and dead cells (Figure 3A bottom two rows and Figures 3B, 3C, 4C, and 4D). The ratio-metric data show that 10-fold more cell death occurs in Phase 1 than in Phase 2, hence, logarithmic ratios of the intensities are provided in Figure 4D to highlight Phase 2 cell death. In all remaining ratio kymographs, we present the log-transformed data. The companion linear ratio data are provided in the Supplemental information.

Phase 1 cell death largely preceded the formation of sectors, which began along the colony rim (Figure 3A, denoted by yellow arrows). The finding that sector initiation sites co-localize with regions of high Phase 1 cell death is notable given that variants could have emerged anywhere in the colony, as has been observed previously, for example, in *Bacillus subtilis* (Hashuel and Ben-Yehuda, 2019). Rather, in *V. cholerae* 2740–80, sectors arise exclusively in regions of high Phase 1 cell death. Furthermore, cells in the sectors were largely living compared to cells in neighboring non-sectored, parental regions of the colony that were undergoing high cell death (Figure 3A and Video S1). This result suggests that the mutations in the arising variant strains suppress the cell death mechanism.

Cell death dynamics were strikingly altered in the T6SS inactive ∆8 strain. Compared to *V. cholerae* 2740–80, Phase 1 cell death along the colony rim was ~2- to 10-fold lower in the ∆8 strain and Phase 2 cell death in the colony interior did not occur (Figures 3B, 3C, and 4E–4H). Despite displaying decreased cell death, the ∆8 strain developed sectors (Figure 3C). We conclude that the T6SS is involved in driving both phases of cell death in *V. cholerae* colonies. Because sectoring was not abolished in the ∆8 strain, mechanism(s) in addition to T6SS can drive sectoring.

### Distinct T6SS effector-immunity protein pairs drive each phase of *V. cholerae* 2740–80 cell death

**Figure 5.**
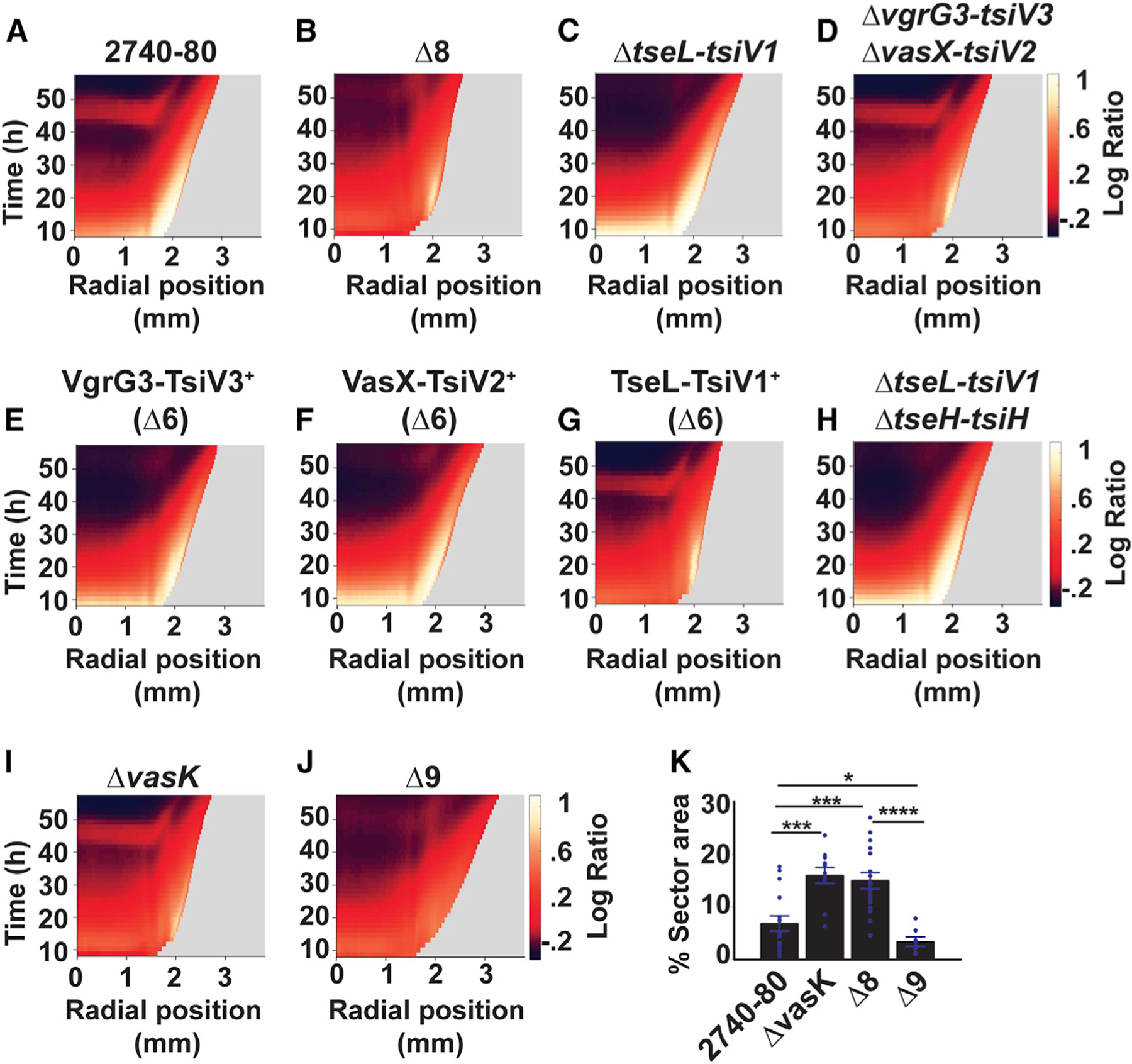
The T6SS apparatus mediates *V. cholerae* colony sectoring, distinct T6SS effector-immunity protein pairs drive each phase of cell death, and Phase 2 cell death does not require the T6SS injection machinery (A–J) Logarithmic ratio kymographs for the indicated strains. Kymograph data treated as described for Figure 4. Companion linear ratio kymographs for (A)–(H) are provided in Figure S4 and those for panels (I) and (J) are in Figure S7. (K) Colony area occupied by sectors in the indicated strains. Kymographs from one colony are representative of results from 3 to 9 colonies for each strain. In (K), data represent average values from biological replicates (n = 7–16), and error bars show SEMs. Statistical significance was calculated using a two-tailed Student’s *t* test with unequal sample variance. Asterisks: *p < 0.05, ***p < 0.005, ****p < 0.0005. See also Figures S4, S5, S6, and S7.

We wondered which of the four T6SS effector-immunity (hereafter E-I) protein pairs causes cell death in *V. cholerae* 2740–80. To identify the pair, we engineered strains lacking one (∆2), two (∆4), or each combination of three (∆6) E-I protein pairs. We monitored cell death in the four ∆2 strains, two ∆4 strains, and four ∆6 strains using time-lapse microscopy, as in Figures 3 and 4. Data for select strains are displayed in Figure 5. Data for the full set, i.e., for each ∆2, ∆4, and ∆6 strain, are displayed in Figure S4 (linear ratio kymographs), Figure S5 (logarithmic ratio kymographs), and Figure S6 (time series projections).

Each ∆2 strain, lacking one E-I protein pair, displayed Phase 1 cell death along the colony rim that was indistinguishable from that of *V. cholerae* 2740–80 (Figures S4A–S4F, S5A–S5F, and S6A). Data from the strain lacking the TseL-TsiV1 pair is provided as the representative of the ∆2 strains in Figure 5C and should be compared to the data in Figures 5A and 5B for *V. cholerae* 2740–80 and the ∆8 strain, respectively. The ∆4 strain lacking both the VgrG3-TsiV3 and the VasX-TsiV2 protein pairs, by contrast, showed the reduced Phase 1 cell death phenotype of the ∆8 strain (Figures 5B, 5D, S4B, S4G, S5B, and S5G, and S6A). Because the ∆2 strains did not display defects in Phase 1 cell death, while the ∆4 strain was impaired for killing, we conclude that the VgrG3 and VasX effector proteins make redundant contributions to Phase 1 cell death. Confirming this assertion, among the ∆6 strains, only two strains, harboring either VgG3-TsiV3 or VasX-TsiV2 as the sole E-I protein pair, displayed Phase 1 cell death patterns like *V. cholerae* 2740–80 (Figures 5A, 5E–5G, S4A, S4H–S4K, S5A, S5H–S5K, and S6A). Consistent with the idea that the remaining two E-I protein pairs, TseL-TsiV1 and TseH-TsiH, are dispensable for driving Phase 1 cell death, the ∆4 strain lacking both of these protein pairs acted like *V. cholerae* 2740–80 with respect to Phase 1 cell death (Figures 5A, 5H, S4A, S4L, S5A, S5L, and S6A). Thus, either the VgG3 or the VasX effector can mediate Phase 1 cell death.

Regarding Phase 2 cell death, which occurs in the colony interior, among the ∆2 strains, cell death was abolished only in the strain lacking TseL-TsiV1, a phenotype mimicking the ∆8 strain (compare data in Figure 5C in the colony interior to that in 5A and 5B and see Figures S4A–S4F, S5A–S5F, and S6B). Further confirming the role of the TseL-TsiV1 protein pair in Phase 2 cell death, only one ∆6 strain, possessing TseL-TsiV1 as the sole E-I protein pair, showed a Phase 2 cell death pattern akin to that of *V. cholerae* 2740–80 (Figure 5A and 5E–5G; S4A, S4H–S4K, S5A, S5H–S5K, and S6B). Thus, the TseL effector protein is required to drive Phase 2 cell death.

### Phase 2 cell death does not require the T6SS injection machinery

T6SS-dependent killing relies on an injection machine to deliver toxic effector proteins into prey cells. To examine whether the T6SS-dependent killing that takes place in the *V. cholerae* 2740–80 colonies requires the T6SS-injection apparatus, we deleted *vasK*, encoding an essential structural component of the injection machine from *V. cholerae* 2740–80 and from the ∆8 strain. Phase 1 killing along the colony rim was diminished in both the ∆*vasK* and the ∆8 strains, and combining the ∆*vasK* and ∆8 mutations (hereafter the ∆9 strain) reinforced the other’s effects, nearly eliminating Phase 1 cell death (Figures 5A, 5B 5I, 5J, S7A–S7D, and S7H). Thus, possession of a functional T6SS-injection machinery contributes strongly to Phase 1 cell death. We offer possibilities that could account for the synergistic effects of the combined ∆*vasK* and ∆8 mutations in the Discussion.

With respect to Phase 2 cell death in the colony interior, the ∆*vasK* strain displayed no defect while Phase 2 cell death did not occur in the ∆8 and ∆9 strains (Figures 5A, 5B, 5I, 5J, and S7I). Because Phase 2 cell death is driven by the TseL effector protein (Figures 5C, 5G and 5H), we conclude that TseL can cause cell death independent of the T6SS-injection apparatus. This experiment does not allow us to distinguish between whether TseL is translocated to target cells via an alternate mechanism or whether TseL-producing cells experience auto-poisoning.

### T6SS-activity drives colony sectoring

To probe whether T6SS activity influences sectoring, we quantified the sectoring phenotypes in the strains under study. *V.cholerae* 2740–80, strains lacking individual or combinations of E-I protein pairs, the ∆8 strain, and the ∆*vasK* strain all made sectors (Figures S6B and S7I). By contrast, the ∆9 strain consistently formed fewer and/or smaller sectors (Figure S7I). Using machine-learning-driven image segmentation, we measured the area occupied by sectors in the ∆9 strain and its progenitors. Sectors in the ∆*vasK* and ∆8 mutants occupied ~2.5-fold more area than did sectors in *V. cholerae* 2740–80 (Figure 5K). Sectors in the ∆9 mutant occupied ~2-fold less area than sectors in *V. cholerae* 2740–80 and ~5-fold less area than in the ∆*vasK* and ∆8 strains (Figure 5K). We conclude that T6SS-killing activity drives colony sectoring. In the Discussion, we present possible explanations for the unexpected finding that the ∆*vasK* and ∆8 mutations each drive increases in sector area occupancy although, when combined, they reduce the colony area occupied by sectors.

### The LuxO driven LCD QS state eliminates colony sectoring through repression of T6SS-dependent cell killing and activation of Vps-dependent T6SS defense

The above results suggest that T6SS plays a key role in causing cell death and colony sectoring. As mentioned, excess Vps can defend against T6SS killing. We know that QS controls both *t6ss* and *vps* expression in *V. cholerae*. This understanding enables us to put forward and test the idea that the QS LCD-locked variants we isolated exhibit both reduced T6SS activity and high Vps production. Together, these altered traits suppress T6SS-dependent killing in the colony, which decreases overall cell death, the consequence of which is prevention of sectoring.

**Figure 6.**
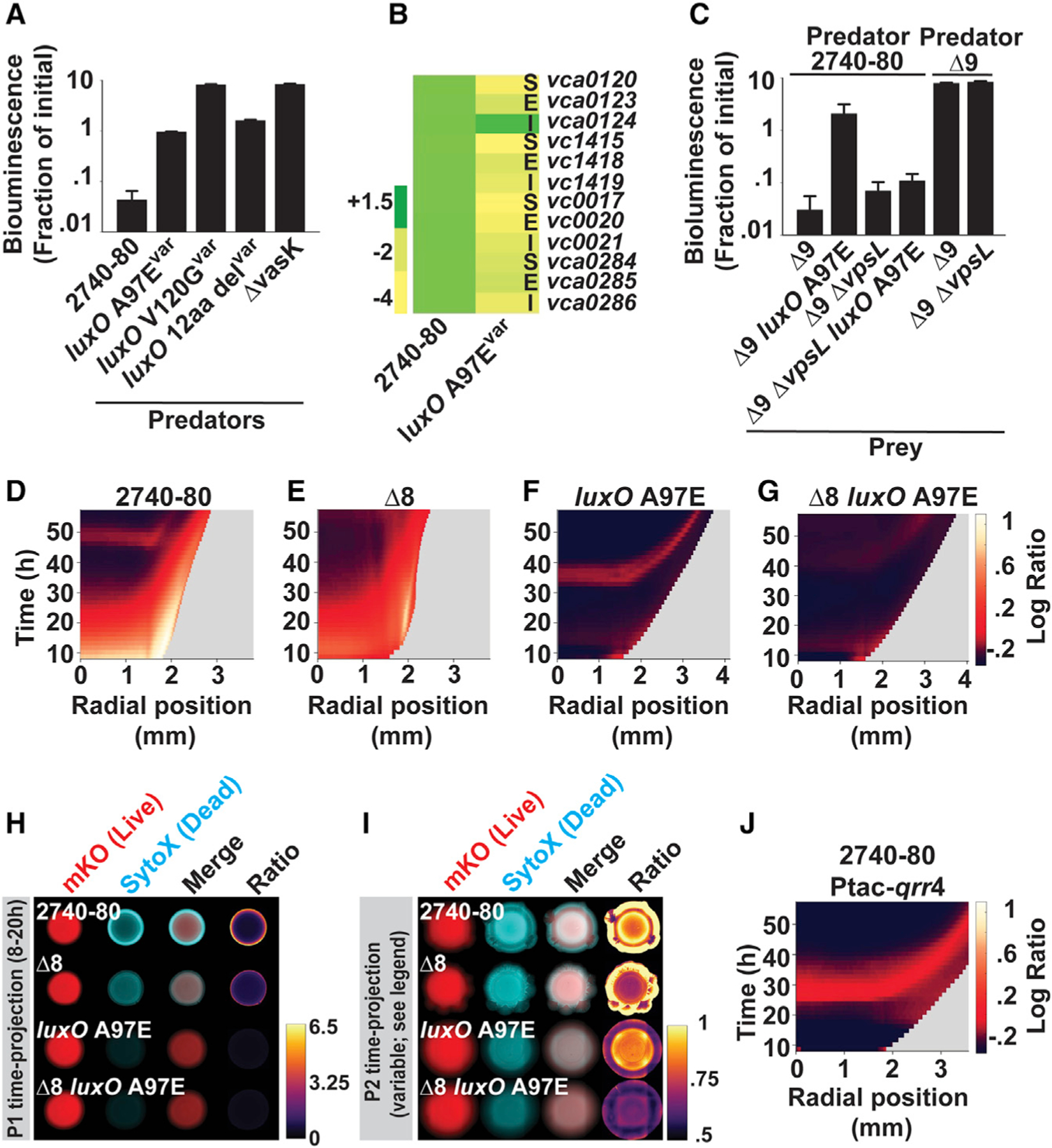
The LCD QS state represses T6SS-dependent killing, activates Vps-dependent T6SS-defense, and eliminates colony sectoring (A) Inter-bacterial T6SS killing assay measuring survival of T6SS-inactive *E. coli* prey following challenge with the indicated *V. cholerae* predator cells. The *E. coli* prey strain constitutively expresses luciferase. Thus, bioluminescence output is a proxy for live cells. (B) Transcript abundance, relative to *V. cholerae* 2740–80, for the indicated strains and genes. The data are color-mapped, and the scale bar displays fold-change. Designations: S = T6SS secretion protein, E = T6SS effector toxin, I = T6SS immunity protein. (C) As in A, measuring survival of the indicated *V. cholerae* prey strains following challenge with the designated *V. cholerae* 2740–80 predator strains. (D–G) Logarithmic ratio kymographs for the indicated strains. (H and I) Time-projections showing cell death and sectoring for the indicated strains and phases. (J) Logarithmic ratio kymograph for *V. cholerae* 2740–80 in which *qrr*4 is overexpressed.. Kymograph data in panels (D)–(G) and (J) treated as described for Figure 3. Kymographs from one colony are representative of results from 3 to 9 colonies for each strain. In (A) and (C), data represent average values from biological replicates (n = 3), and error bars show SDs. In (B), average values were obtained from three biological replicates and two technical replicates for each strain (n = 6). In (I), due to differences in timing of Phase 2 cell death among strains (compare Phase 2 initiation times in [D] and [F]), time-projections for *V. cholerae* 2740–80 and the ∆8 strain show data from 42.5 to 56 h, while for the *luxO* A97E and ∆8 *luxO* A97E strains, data are shown from 30.5 to 44 h. See also Figures S2, S3, and S7.

To assess whether the LCD QS state alters *V. cholerae* 2740–80 T6SS activity, we measured the capacity of the *luxO* variants we isolated to kill *Escherichia coli* in an inter-bacterial T6SS-dependent killing assay. As prey, we used an *E. coli* strain that constitutively produces *lux*, and thus, light output tracks with live prey cells. When the *luxO* variants were used as predators, there was a 10- to 100-fold decrease in prey killing relative to when *V.cholerae* 2740–80 was predator (Figure 6A). No killing occurred when the ∆*vasK* strain was the predator, confirming that killing requires T6SS activity (Figure 6A). To verify that the decreases in killing ability of the *luxO* variants were a consequence of decreased expression of *t6ss* genes, in one representative *luxO* variant (*luxO* A97E), we quantified transcript levels for the genes specifying each E-I protein pair and select genes encoding T6SS structural components. Figure 6B shows the results. Compared to *V. cholerae* 2740–80, the *luxO* A97E variant exhibited 2- to 4-fold decreased expression of every tested *t6ss* gene. Thus, LuxO-driven LCD behavior suppresses T6SS-killing activity in *V. cholerae* 2740–80.

We examined whether increased Vps production boosts the defense capacity of the *V. cholerae* 2740–80 LCD-locked QS variants against incoming T6SS attacks. We already know that all the *V. cholerae* 2740–80 LCD-locked QS variants exhibit increased expression of *vps* (Figure 2D), so we again used the *luxO* A97E allele as our representative for this analysis. To do the experiment, we introduced the *luxO* A97E and ∆*vpsL* mutations, both alone and in combination, into the ∆9 strain. This strategy allowed us to avoid possible complications from secondary mutations that might be present in the original *luxO* A97E variant strain. Moreover, because each strain in this set lacks all T6SS-immunity proteins, they are susceptible to T6SS-dependent killing following challenge with *V. cholerae* 2740–80. Lastly, all prey strains were also engineered to carry a constitutive *lux* reporter enabling tracking of survival. Relative to the ∆9 strain, the ∆9 *luxO* A97E strain displayed ~100-fold increased survival against the *V. cholerae* 2740–80 predator while the ∆9 ∆*vpsL* and ∆9 ∆*vpsL luxO* A97E strains showed no survival enhancement (Figure 6C). No killing occurred when the ∆9 or ∆9 ∆*vpsL* strains were challenged with the ∆9 strain as predator, again confirming that T6SS activity drives killing in our assay (Figure 6C). Thus, in the *luxO* A97E LCD-locked variant, and presumably the other variants we isolated, increased Vps production driven by QS functioning in the LCD-mode promotes enhanced defense against incoming T6SS attacks. Moreover, the results show that the protective effect of high level Vps production can overcome the sensitivity to killing caused by complete lack of immunity factors.

Beyond effects on T6SS-killing and T6SS-defense, we examined whether the LCD-locked QS mode promotes an altered cell death pattern. Here, we again used the *luxO* A97E allele as the representative, and as above to avoid complications from possible secondary mutations in the original variant, we reconstructed all needed mutations in *V. cholerae* 2740–80 and in the ∆8 strain. Phase 1 cell death along the colony rim was abolished in the *luxO* A97E and ∆8 *luxO* A97E strains (Figures 6D–6H, S3F–S3J, S7A, S7B, S7E, and S7F). Indeed, the *luxO* A97E mutation eliminated the residual Phase 1 cell death that occurs in the ∆8 strain (Figures 6E–6G). Thus, QS fully controls Phase 1 cell death. Given that elimination of T6SS does not abolish all Phase 1 cell death in the ∆8 strain (Figure 6E), another QS-controlled process must be involved in Phase 1 cell death. By contrast, the *luxO* A97E strain displayed Phase 2 cell death in the colony interior like *V. cholerae* 2740–80 (Figures 6D, 6F, and 6I). The ∆8 *luxO* A97E strain mimicked the ∆8 strain and displayed no Phase 2 killing (Figures 6E–6G and 6I). Thus, Phase 2 cell death, which is TseL-dependent (Figures 5C, 5G, and 5H), is not subject to QS regulation. Finally, none of the strains carrying *luxO* A97E sectored, including in the ∆8 background (Figure 6I; see bottom two rows). Thus, the LCD QS state is epistatic to T6SS with respect to sectoring. Because the LCD-locked variants do not sector and have no Phase 1 cell death, but undergo Phase 2 cell death, we infer that Phase 1 cell death is key to the sectoring phenotype whereas Phase 2 cell death may be dispensable for sectoring.

To pinpoint the mechanism that connects QS to T6SS-driven cell death, Vps, and sectoring phenotypes, we focused on the Qrr sRNAs that repress translation of the large *t6ss* gene cluster, and indirectly activate *vps* gene expression (Figure 1) (Shao and Bassler, 2014). To test if the QS phenotypes hinge on Qrr activity, we constitutively expressed one of them, *qrr*4 (Ptac-*qrr*4), in *V. cholerae* 2740–80 and examined the phenotypic consequences. Introduction of Ptac-*qrr*4 abolished Phase 1 cell death and sectoring in *V. cholerae* 2740–80, including the low-level cell death that occurs in the ∆8 strain (Figure 6J and S7G). Phase 2 cell death occurred (Figure 6J). Thus, overexpression of Qrr4 is sufficient to mimic the phenotype caused by the *luxO* A97E mutation (Figures 6F and 6J). We conclude that in our LCD-locked *luxO* QS variants, it is the Qrr sRNAs that repress T6SS components and activate Vps production. Together, these changes lower cell death and abolish sectoring.

### *V. cholerae* 2740–80 exhibits T6SS- and Vps-dependent inhibition of growth of neighboring kin cells

Our data showing that possession of a functional T6SS-injection machine contributes strongly to Phase 1 cell death and that T6SS-killing activity is suppressed by the Vps matrix (Figure 6) suggest that *V. cholerae* 2740–80 cells in colonies kill neighboring kin cells (Figure 5). To test this prediction, we engineered two strains; *V. cholerae* 2740–80 ∆*vpsL* and *V. cholerae* 2740–80 ∆*vasK*, each carrying a constitutive *lux* reporter to enable tracking of cell survival. Each of these luciferase-containing strains was mixed with the non-bioluminescent version of itself. This strategy allowed the bioluminescent strain to be the prey and the otherwise isogenic non-bioluminescent strain to be the predator. For the reference predator-prey control pair, we like-wise mixed *V. cholerae* 2740–80 with *V. cholerae* 2740–80 *lux*. Relative to the control, *V. cholerae* 2740–80 ∆*vpsL lux* showed the lowest survival and *V. cholerae* 2740–80 ∆*vasK lux* displayed the highest survival (Figure S2B). Thus, when it possesses a functional T6SS machine, *V. cholerae* 2740–80 inhibits growth of its nearby genetically identical kin. However, the presence of Vps protects against kin-killing. These findings further confirm the results shown in Figure 6.

### *V. cholerae* 2740–80 colonies exhibit spatial-temporal patterns of QS, T6SS-offense, and T6SS-defense gene expression

Our data suggest that cell death patterns and sectoring in *V. cholerae* colonies arise because of spatiotemporal changes in QS, T6SS killing activity, and Vps-mediated defense against T6SS injection. To verify this notion, and as a companion to our activity analyses, we measured transcript levels of QS, T6SS, and Vps production genes in cells obtained from the rims and centers of *V. cholerae* 2740–80 colonies to determine if they too show particular spatial patterns. The experiment was conducted at 20 h of growth, when *V. cholerae* 2740–80 colonies are undergoing Phase 1 cell death along the colony rim (Figure 4). As a control, we performed identical analyses with cells obtained from colonies of the *V. cholerae* 2740–80 *luxO* A97E strain that is LCD-locked and shows no Phase 1 spatial-temporal cell death pattern (Figure 6F).

**Figure 7.**
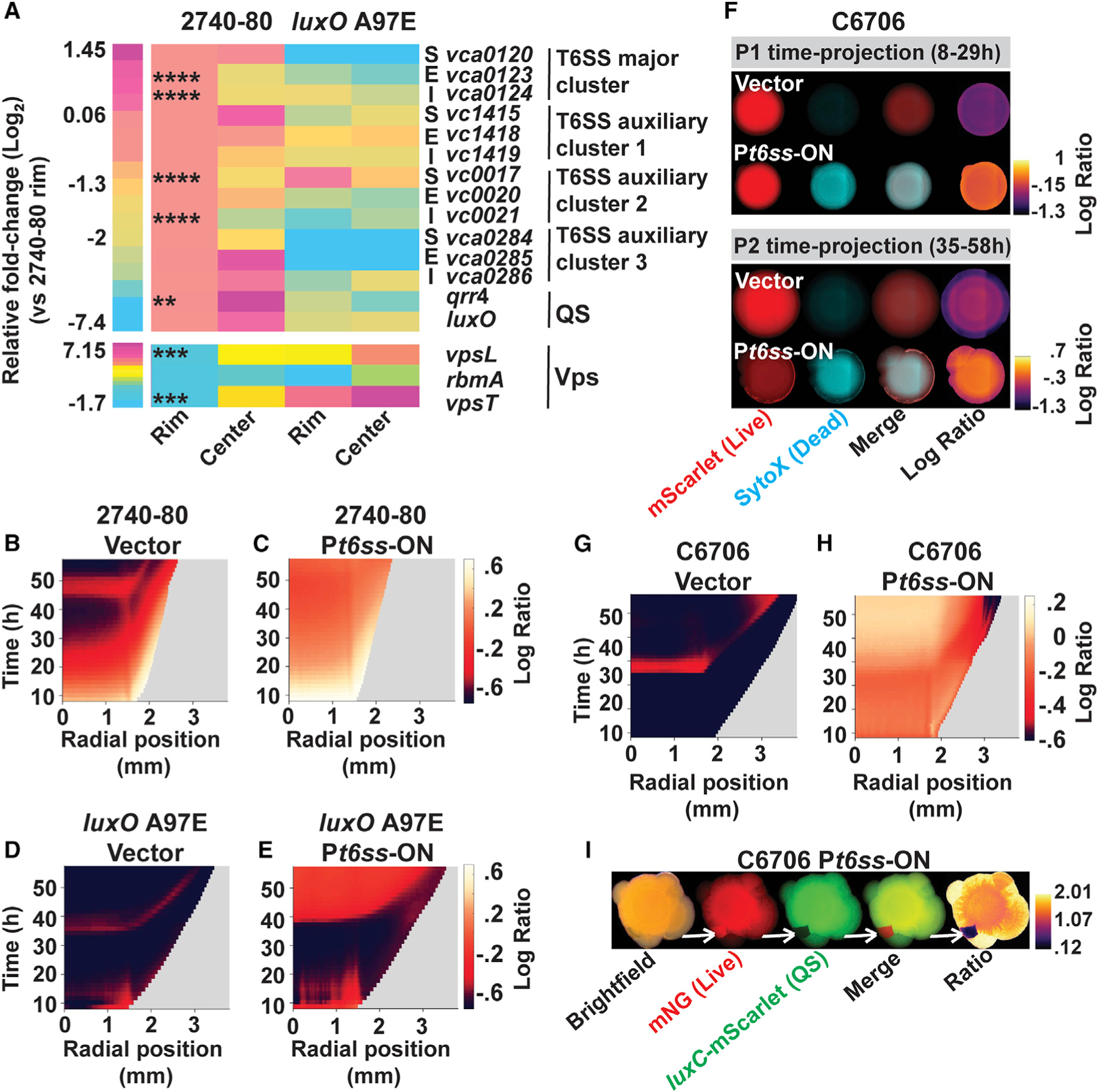
QS, *vps*, and *t6ss* gene expression patterns are spatially distinct in colonies and alteration of regional expression by inducible *t6ss* expression eliminates cell death patterns in *V. cholerae* 2740–80, and moreover, drives sectoring and the emergence of QS variants in the normally T6SS-silent *V. cholerae* C6706 strain (A) Spatial expression of *t6ss*, QS, and *vps* genes in colonies of *V. cholerae* 2740–80 and *V. cholerae* 2740–80 *luxO* A97E. Transcript abundances of the indicated genes in cells isolated from the designated colony areas, measured by qRT-PCR. Data are color-mapped, and the scale bar shows Log_2_ fold-changes relative to that at the colony rim of *V. cholerae* 2740–80. Note that different scales are used for the two heatmaps. Designations: S = T6SS secretion protein, E = T6SS effector protein, I = T6SS immunity protein. Regarding the rim: cells were isolated from two regions per colony and samples from four colonies were combined to yield the final sample. Regarding the colony center: cells were isolated from four colonies and combined to yield the final sample. In each case, rim and center, three such samples were prepared representing cells from twelve colonies. The color-mapped data represent the average values from these three samples, assayed in technical replicates (n = 6). Cells were isolated at 20 h post inoculation, a time when only Phase 1 cell death occurs (Figure 4). (B–E) Logarithmic ratio kymographs for the indicated strains carrying the designated plasmids. (F) Time-projections showing cell death and sectoring in *V. cholerae* C6706 carrying the indicated plasmids (G and H) Logarithmic ratio kymographs for the indicated strains. (I) Stereo-microscope images of *V. cholerae* C6706 carrying P*t6ss*-ON, constitutively producing mNeonGreen (denoted: mNG) to mark live cells, and a QS-activated reporter (*luxC*-mScarlet) following three days of growth. White arrows mark a sector that contains live cells but exhibits little *luxC*-mScarlet activity. Kymograph data in panels (B)–(E) and (G) and (H) treated as described for Figure 4. Kymographs from one colony are representative of results from 3 to 9 colonies for each strain. Note: because plasmid-driven production of T6SS components drives large increases in cell death, the ratio time-projection images in (F) are displayed on log scales, unlike those in Figures 3 and 6, which are shown on linear scales. Statistical significance, displayed for select genes of interest in (A), was assessed by comparison between *V. cholerae* 2740–80 transcript levels at the rim compared to the center for the same strain and calculated using a two-tailed Student’s t test with unequal sample variance. Asterisks: *p < 0.05, **p < 0.01, ***p < 0.005, ****p < 0.0005. See also Figure S8.

Regarding *V. cholerae* 2740–80 colonies, expression of genes located in the major *t6ss* gene cluster and auxiliary cluster 2 were ~2- to 5-fold lower in the colony center than at the rim where cell death takes place (Figure 7A). Gratifyingly, these two clusters include the *vgrG*3-*tsiV3* and *vasX*-*tsiV2* genes encoding the E-I protein pairs which we demonstrated mediate Phase 1 cell death (Figures 5D–5F). Gene expression from auxiliary clusters 1 and 3 was unchanged or only modestly altered between the rim and interior (Figure 7A). By contrast, expression of the QS *qrr*4 gene, encoding the Qrr4 sRNA repressor of *t6ss* genes (Figure 6J), and expression of genes involved in Vps production, which defend against incoming T6SS attacks (Figure 6C), were ~3- to 6-fold higher in cells in the colony center than at the rim (Figure 7A). To accompany these results, in Figures S8H and S8I, a QS-controlled transcriptional reporter (*luxC-*mNeonGreen) is quantified in colonies of *V. cholerae* 2740–80 and shows that HCD QS activity is higher along the rims than in the centers of colonies during Phase 1 cell death.

Regarding *V. cholerae* 2740–80 *luxO* A97E colonies, irrespective of location in the colony, expression of each measured *t6ss* gene was ~3- to 20-fold lower than in cells from *V. cholerae* 2740–80 (Figure 7A). Levels of the *qrr*4 transcript were lower throughout the *V. cholerae* 2740–80 *luxO* A97E colony despite ~100-fold induction of the *qrr*4 promoter in this strain (Figure 2B). This result is consistent with previous studies showing that in LCD-locked *V. cholerae* strains, interaction of target mRNAs with the Qrr4 sRNA promotes degradation of Qrr4 (Lenz et al., 2004). Lastly, relative to *V. cholerae* 2740–80, expression of Vps production genes at the colony rim were ~2- to 30-fold higher, and at the colony center, they were up to ~150-fold higher in the *V. cholerae* 2740–80 *luxO* A97E strain (Figure 7A).

We conclude that during Phase 1 cell death in *V. cholerae* 2740–80, genes involved in HCD QS and T6SS-killing are expressed at higher levels in cells residing along the rim of the colony than in the center, while cells in the center of the colony express higher levels of genes specifying LCD QS behaviors and T6SS-defense traits (i.e., Vps) than cells at the rim. We propose that these spatially distinct gene expression patterns drive the cell death patterns that occur in *V. cholerae* 2740–80 colonies. Presumably, differential expression of the same genes also occurs in a temporally distinct manner, as suggested by our data in Figures 4 and 5, and in Figures S8H and S8I, but that feature remains to be verified.

### Constitutive expression of *t6ss* genes eliminates the spatiotemporal cell death patterns in *V. cholerae* 2740–80 and restores cell death in the LCD-locked QS strain

Two predictions arise from our findings that QS, T6SS, and Vps genes are expressed in particular spatial, and presumably temporal, patterns in *V. cholerae* 2740–80 colonies. First, forcing production of T6SS machinery in all cells in *V. cholerae* 2740–80 colonies would cause cell death across the entire population and eliminate any spatiotemporal pattern. Second, re-establishment of T6SS production in a LCD-locked QS strain would restore cell death and drive sectoring. To test the first prediction, we introduced a plasmid carrying the T6SS activators *qstR* and *tfoX* under control of an arabinose inducible promoter (called P*t6ss*-ON) into *V. cholerae* 2740–80 (Bernardy et al., 2016; Jaskólska et al., 2018; Metzger et al., 2016). To test the second prediction, we did the same experiment in the LCD-locked *luxO* A97E strain. In each case, we monitored cell death and sectoring.

Induction of P*t6ss*-ON-driven T6SS production caused a 10- to 40-fold increase in cell death in *V. cholerae* 2740–80 compared to the strain carrying the empty vector (Figure 7B, 7C, S8A, and S8B). Notably, *V. cholerae* 2740–80 harboring the empty vector displayed the characteristic Phase 1 and Phase 2 cell death patterns, while introduction of P*t6ss*-ON caused cell death across the colony (Figures 7B and 7C). Thus, the normal pattern of cell death that occurs in *V. cholerae* 2740–80 colonies is a consequence of non-homogeneous *t6ss* expression and the ensuing non-homogeneous T6SS activity.

Introduction of P*t6ss*-ON into the *luxO* A97E strain caused little cell death until ~40 h, after which cell death became detectable and occurred homogenously across the colony interior (Figures 7D–7E, S8A, and S8B). Once cell death commenced, the level was roughly the same as that in *V. cholerae* 2740–80 carrying P*t6ss*-ON. We do not yet understand how repression of cell death is relieved after 40 h in the *luxO* A97E LCD-locked QS strain. Possibilities include spatial/temporal changes in expression of T6SS-defense genes (Vps) or QS genes (Qrr4) that allow T6SS-killing to occur during later growth times.

Regarding sectoring, *V. cholerae* 2740–80 carrying P*t6ss*-ON formed sectors, whereas only minimal sectoring occurred following introduction of P*t6ss*-ON into the *luxO* A97E strain, visible as radial streaks (Figure S8B. and see also enlarged images in Figure S8C). We do not understand why sectoring was not fully restored. Likely, plasmid expression of the genes encoding the two T6SS activators does not perfectly mimic native control of the entire set of *t6ss* gene clusters. Nonetheless, we conclude that QS governs the region-specific expression of Phase 1 T6SS activity, thereby driving cell death and sectoring.

### T6SS-dependent cell death, sectoring, and emergence of QS variants occurs when *t6ss* genes are expressed in a normally T6SS-silent *V. cholerae* strain

To garner additional evidence demonstrating that both cell death and sectoring are T6SS-dependent in *V. cholerae*, we used our P*t6ss*-ON construct to induce *t6ss* gene expression in *V. cholerae* C6706, which, as mentioned, does not express *t6ss* genes under laboratory growth conditions and does not sector (Figure 2A). We assessed the consequences to cell death and sectoring. *V. cholerae* C6706 carrying the empty vector displayed no Phase 1 cell death (Figures 7F, top row, and 7G). There was modest Phase 2 cell death, but notably, ~10-fold lower than that in *V. cholerae* 2740–80 (compare data in Figure 7G to that in Figure 7B). Figures 7F–7H show that P*t6ss*-ON-driven T6SS production increased cell death ~10-fold in *V. cholerae* C6706. Cell death occurred across the entire colony, consistent with homogeneous expression of *t6ss* genes. Furthermore, sectors formed with timing similar to that in *V. cholerae* 2740–80 (Figure 7F, bottom row). Thus, high T6SS activity causes cell death and the appearance of sectors in both pandemic (*V. cholerae* C6706) and pre-pandemic (*V. cholerae* 2740–80) *V. cholerae* strains.

To discover whether the *V. cholerae* C6706 T6SS-dependent sectors are enriched in cells with altered QS behaviors, we imaged sectors in a *V. cholerae* C6706 strain harboring P*t6ss*-ON, a constitutively produced fluorescent reporter marking live cells (mNeonGreen), and a QS-activated-fluorescent reporter (*luxC*-mScarlet). In ~5%–10% of the sectors, the live cells present did not express the QS reporter, indicating that the cells in these sectors had acquired mutation(s) that result in LCD-locked QS behavior (Figure 7I; indicated with white arrows). We conclude that T6SS killing activity in *V. cholerae* colonies imposes a selective pressure to acquire LCD-locked QS mutations, presumably enhancing growth and promoting sector formation.

## DISCUSSION

Here, we discover that QS-controlled T6SS-mediated cell death provides a selective pressure that allows QS-defective strains of *V. cholerae* to arise that are capable of evading T6SS-killing. T6SS-mediated cell death occurs in a two-phase, spatiotemporal manner. Distinct T6SS effectors, VgrG3 and VasX for Phase 1 and TseL for Phase 2, are required for killing. QS controls Phase 1 cell death and, indeed, the underlying QS, *t6ss*, and *vps* genes show regional differences in expression during Phase 1 cell death. Phase 1 cell death is key for sectoring to occur and thus for enhanced genetic diversity to arise in the population (see model in Figure S7J).

Our findings reveal an unanticipated facet of *V. cholerae* T6SS biology: the *V. cholerae* T6SS machinery, which was understood to deliver toxins to non-kin cells, can be deployed to eliminate sibling cells. Thus, the T6SS may have unappreciated roles in intra-specific antagonism. It was surprising that sibling cells succumb to incoming T6SS attacks given that they produce T6SS-effector neutralizing immunity proteins. One previous example of T6SS-dependent kin-killing has been reported in *Myxococcus xanthus*, in which slow-growing or auxotrophic cells in the population exhibit reduced T6SS protein production, including T6SS immunity proteins, rendering them susceptible to killing by faster growing nearby cells that produce higher levels of T6SS toxins (Troselj et al., 2018). The Troselj et al. work provides clues to a potential biological rationale for kin-killing in *V. cholerae* colonies. It is known that cells residing in colonies compete for limited resources, including space and nutrients. One would expect such competition to be most fierce in mature/aged colonies, which is when cell death occurs in *V. cholerae* colonies. It could be that cells with superior fitness engage in cannibalism. They eliminate less-fit cells in the colony, and in so doing, acquire resources formerly used by (space) or released from (nutrients) the dead cells. One prediction of this notion is that kin-killing would be suppressed when nutrients are plentiful. Indeed, we find that growth in resource-rich nutrient broth suppresses cell death and sectoring (Figures S8D–S8G).

*V. cholerae* colonizes chitinous surfaces in its marine environment, and chitin acts as a cue that activates *t6ss* gene expression (Borgeaud et al., 2015; Meibom et al., 2005). Curiously, clinical and environmental isolates of *V. cholerae* harbor QS-inactivating mutations at a high frequency (Joelsson et al., 2006). Indeed, interrogation of the QS function of 16 *V. cholerae* strains revealed that half of the surveyed strains possess dysfunctional QS systems that make the strains display QS LCD-type behaviors. Likewise, among the three original *V. cholerae* isolates used in our study, two of the strains, *V. cholerae* V52 and *V. cholerae* 2740–80, have QS systems that vary in function from the norm (Figures S2C–S2E). The mechanism driving the high frequency emergence of QS-dysfunctional strains of *V. cholerae* has remained mysterious. Our results demonstrate that during laboratory growth, T6SS-killing fosters the emergence of variants with altered QS function in *V. cholerae* colonies. Given that the T6SS machinery is induced on chitinous surfaces, we propose that T6SS-driven kin-killing likely also occurs in natural habitats and perhaps during disease, and this mechanism propels genetic diversity. It is also intriguing that the arising variant strains exhibit a range of T6SS activity levels and/or capacities to neutralize incoming T6SS attacks (Figures 6A, 6C, and S2F). Such a possibility also exists for the other two variants recovered in our suppressor screen, which acquired mutations affecting CspA and PyrG. CspA, a cold shock protein, modulates T6SS killing activity, while the PyrG cytidine synthase likely influences T6SS function by altering levels of cytidine, a ligand for the CytR transcription factor that activates *t6ss* genes and represses biofilm formation genes (Barbier et al., 1997; Townsley et al., 2016; Watve et al., 2015). Thus, T6SS-driven intra-specific antagonism selects for acquisition of mutations in QS and other pathways that modify expression of *t6ss* offensive and defensive genes in *V. cholerae*. This mechanism could enable iterative improvements in tuning of T6SS activity to various niches. Indeed, this notion is best illustrated by HapR A52T (Figures 2B–2D and S2F) which has distinct effects on different HapR target promoters. Pertinent to our study, the strain carrying HapR A52T does not repress *vps* genes, exhibits increased T6SS activity, and displays wildtype HapR behavior in driving QS genes. Thus, *V. cholerae* 2740–80 *hapR* A52T may be ‘‘optimized’’ with respect to its balance between offensive and defensive capacities, while continuing to be able to communicate with its bacterial neighbors.

We discovered that T6SS-driven Phase 1 cell killing relies on the T6SS VasK-dependent injection machinery (Figure 5I). Curiously, combining the ∆8 mutation, which eliminates all effectors, with the ∆*vasK* mutation, eliminating the injection apparatus, had a modest additive effect with respect to cell death (additivity is best visualized in Figures S7B–S7D). Two possible explanations occur to us. First, despite lacking T6SS toxins, the ∆8 strain nonetheless possesses an intact T6SS injection machine. It is possible that a subset of cells in the colony have damaged cell envelopes, rendering them susceptible to harm upon physical penetration by the T6SS needle, which is expelled with considerable energy into target cells (Kamal et al., 2020; Wang et al., 2019). Emphasizing this line of thought, a recent study found that *V. cholerae* cells possessing only the injection machinery, but no effectors, can inhibit the growth of *Pseudomonas aeruginosa* strains lacking the TolB protein, which is important for maintaining outer membrane integrity (Kamal et al., 2020). A second possibility is that the *V. cholerae* ∆8 strain continues to synthesize an as-yet-unidentified effector toxin that employs the T6SS injection apparatus for its killing activity.

Phase 2 cell killing required the T6SS TseL effector toxin but not the T6SS injection machinery (Figures 5C and 5I). It is currently unclear whether TseL causes self-killing or if it can be secreted via an alternate secretion mechanism. In support of the notion that TseL contributes to self-killing, Ho et al. (2017) showed that TseL can be trafficked from the cytosol to the periplasm via a non-T6SS-dependent mechanism. Thus, one possibility is that time- and region-specific trafficking of TseL to the periplasmic compartment promotes Phase 2 cell death. TseL is a phospholipase (Dong et al., 2013). An alternative possibility is that TseL residing in the cytoplasm destroys essential cytoplasmic factor(s), such as precursors in phospholipid biosynthesis, the absence of which would cause cell death.

In multicellular organisms, including humans, key segments of development rely on genetically regulated and time- and region-specific cell death processes (Fink and Cookson, 2005; Kerr et al., 1972). In a striking parallel, we show here that cell death in *V. cholerae* colonies is QS-regulated and occurs in a time- and region-specific manner. Cell death wave(s) were recently reported to guide eukaryotic apoptosis in *Xenopus laevis* (African frog) eggs (Cheng and Ferrell, 2018). Our time-lapse videos and kymograph analyses hint that in *V. cholerae*, cell death during Phase 2 may also propagate as a wave (see especially Video S1). Although it is currently speculative, if correct, this feature would mirror what occurs in eukaryotes. We are currently exploring the origin of the wave-like behavior observed here.

Beyond the *V. cholerae* cell death patterns revealed here, recent studies show that other bacterial communities also display patterns. For example, in *B. subtilis* colony biofilms, genes involved in the nitrogen stress response are expressed in a concentric ring-like pattern. Intriguingly, similar to what we show here, the *B. subtilis* patterns occur in mature colonies and nutrient levels are key (Chou et al., 2022). Cells in *P. aeruginosa* colony biofilms are reported to organize into concentric ring-like zones with each ring displaying a different metabolic capacity. Ring formation is controlled by light and temperature stimulation (Kahl et al., 2022).

Regarding the sequential timing of the two phases of cell death in *V. cholerae* 2740–80, we note that Phase 2 cell death commences only after Phase 1 death subsides. Also, in QS LCD-locked strains (*luxO* A97E or *V. cholerae* 2740–80 carrying Ptac-*qrr*4), which lack Phase 1 cell death, the timing of onset of Phase 2 cell death shifts dramatically, initiating ~24 h earlier than in a strain that is wildtype for QS (compare timing in Figure 6D to that in Figure 6J). Thus, it appears that the timing and occurrence of Phase 1 killing sets the timing of Phase 2 killing. Possibly, Phase 1 killing, which occurs at the colony rim among the youngest members of the colony, functions to delay cell death in the population elders; as Phase 2 killing occurs in the colony interior which contains the oldest cells in the colony. Possibly, cells undergoing death at the colony rim release a ‘‘defer/delay’’ signal that is detected by cells in the colony interior. By alerting older cells to impending cell death, such a signal could function to buy them time to protect themselves. If so, such a scenario would present another fascinating parallel to eukaryotic cell death where, following initiation of apoptosis, dying cells release chemical signals that are detected by stem cells, prompting the stem cells (which are the oldest cells in eukaryotic tissue communities) to mount defenses that ensure their survival and, in turn, their capacity for future tissue re-population (Xing et al., 2015).

### Limitations of the study

Our work shows that QS and T6SS mediate spatiotemporal cell death in *Vibrio cholerae* and Phase 1 cell death is underpinned by regionally distinct patterns of expression of QS, *t6ss* and *vps* genes. It would be fascinating to assess gene expression patterns and production/activities of QS, Vps, and T6SS components at the level of individual cells. However, currently imaging on agar surfaces precludes such single cell level analyses. In the future, when technologically feasible, gene expression and cell death patterns should be quantified with single cell resolution in *V. cholerae* colonies. Our findings in Figures 7A and 7I and Figures S8H and S8I show that community-wide gene expression occurs in spatially heterogeneous patterns. Our initial explorations suggest that gene expression patterns are altered temporally (Figures S8H and S8I), but this point remains to be tested rigorously.

## STAR★METHODS

### RESOURCE AVAILABILITY

#### Lead contact

Further information and requests for resources and reagents should be directed to and will be fulfilled by Dr. Bonnie L. Bassler (bbassler@princeton.edu).

#### Materials availability

Strains and reagents used in this study are available upon request from Dr. Bonnie L. Bassler.

#### Data and code availability

- Data reported in this study will be shared by the lead contact upon request.
- Original code used in this study has been deposited at Zenodo and is publicly available as of the date of publication. DOIs are listed in the key resources table.
- Any additional information required to reanalyze the data reported in this paper is available from the lead contact upon request.

#### EXPERIMENTAL MODEL AND SUBJECT DETAILS

##### Bacterial growth

*Saccharomyces cerevisiae* and *E. coli* Top10 were used for cloning. *E. coli* S17–1 λ*pir* was used for conjugations. Cultures of *V. cholerae* and *E. coli* were grown in LB medium at 37°C with shaking, with a headspace to growth medium volume ratio of 7. The only exception is that prey strains for killing assays were grown overnight at 30°C. When required, media were supplemented with streptomycin, 200 μg/mL; kanamycin 50 μg/mL; polymyxin B, 50 μg/mL; chloramphenicol, 1 μg/mL; spectinomycin, 200 μg/mL. In experiments requiring induction of gene expression, all media used were supplemented with 0.1% arabinose. All *V. cholerae* assays were performed at 30°C unless otherwise noted. LB medium (both liquid and solid) was prepared with dd H_2_O, 100% Tap H_2_O, or 80% Tap and 20% dd H_2_O. Changes in media preparation were a consequence of COVID-imposed supply issues and LB reagent acquired from multiple suppliers. Differences in batches affected the timing of assays and amount of sectoring. Consistent phenotypes could be obtained when solid LB medium was prepared with 80% Tap and 20% dd H_2_O and liquid LB medium was prepared with 100% Tap H_2_O. Bioluminescence-reporter assays were conducted as previously described (Mashruwala and Bassler, 2020). Where indicated, relative light units (RLU) denote bioluminescence output divided by the culture optical density.

##### Strain construction

Chromosomal alterations in *V. cholerae* strains were introduced using the pRE112 suicide vector harboring the counter-selectable *sacB* gene as previously described (Edwards et al., 1998; Eickhoff et al., 2021). All strains used in the study are listed in the Key Resources Table. Unless otherwise specified, chromosomal DNA from *V. cholerae* 2740–80 was used as template for PCR reactions. Plasmids were constructed using pBAD-pEVS or pRE112 as backbones and assembled using enzyme-free XthA-dependent *in vivo* recombination cloning or yeast-recombination-assisted assembly as previously described (Beyer et al., 2015; Joska et al., 2014; Mashruwala and Boyd, 2016; Nozaki and Niki, 2018). Plasmids were introduced into *V. cholerae* strains by conjugation with *E. coli* S17–1 λ*pir*. Plasmids used in this work are listed in the Key Resources Table.

#### METHOD DETAILS

##### Materials

Kits for gel purification, plasmid-preparation, RNA-preparation (RNeasy), qRT-PCR, and RNA-Protect reagent were purchased from Qiagen. iProof DNA polymerase and deoxynucleoside triphosphates were purchased from Biorad.

##### Colony sectoring and cell death assay

A 700 μL aliquot of an overnight culture of *V. cholerae* was combined with 4 mm glass beads in an Eppendorf tube and subjected to vortex for 5 min to disperse aggregates. The culture was diluted with PBS to a final OD_600_ of 0.5. The sample was again subjected to vortex, without glass beads, for 5 min. A 1 μL aliquot of this suspension was spotted onto 35 mL of solid LB agar in a one well plate and allowed to dry for 5 min at room temperature. The plate was incubated for the remainder of the assay at 30°C. Up to 24 such samples were aliquoted onto each agar pad. Sector formation became visible between 18 and 48 h. When required, the LB agar medium was supplemented with 2 μM SytoX dye (ThermoFisher) (Asally et al., 2012).

##### Bioluminescence-based T6SS-dependent inter-bacterial killing assay

Prey cells constitutively expressed the *luxCDABE* operon, incorporated onto the chromosome (*V. cholerae*) or a plasmid (*E. coli* Top10). Prey cell light production was quantified to track surviving cells. To initiate the killing assay, 800 μL of overnight cultures of prey and predator strains were concentrated 2-fold by centrifugation and resuspension in 400 μL PBS. The predator cell suspension was combined with 4 mm glass beads in an Eppendorf tube and subjected to vortex for 5 min to disperse aggregates. In experiments in which effects of Vps production on T6SS-driven killing were examined, rather than apply vortex, cells were gently resuspended with a pipette to preserve biofilm structures. In the case of prey, cultures were divided in half. One-half was subjected to vortex, as described above, and used to obtain the OD_600_ measurement. The other half of the culture was used as the prey cells. Predator and prey suspensions were diluted to a final OD_600_ of 3 with PBS. Subsequently, 4 μL of prey cell suspension were combined with 16 μL of predator cell suspension and subjected to gentle pulse-vortex to mix. 1 μL of such cell suspensions were applied in a 12 × 8 grid arrangement in a one-well plate containing 35 μL of LB agar. Up to 24 samples were spotted onto the agar in each one well plate. Samples were allowed to dry for 5 min at room temperature. Subsequently, the plate was incubated in a Biospa Automated Incubator (Biotek) at 30°C and the bioluminescence from prey cells was quantified over time using a Synergy *Neo*2 plate reader (Biotek). Under these assay conditions, maximal T6SS-driven killing of the ∆8 prey strain occurred at ~125 min (Figure S2G). Data from this time-point are presented in the bar graphs in Figures 6A and 6C.

##### Whole genome sequencing and variant calling

*V. cholerae* strains were diluted from freezer stocks into 3 mL of LB medium and cultured for 3–6 h until turbidity occurred (OD_600_ = 1–2). The cells were collected by centrifugation and DNA was purified from them using the DNeasy Blood and Tissue kit (Qiagen, Germany). Subsequently, the DNA was processed and sequenced. Variant calling to identify SNPs of interest was performed by the Microbial Genome Sequencing Center (Pittsburgh, PA). *V. cholerae* N16961 was used as the reference genome.

##### RNA isolation and quantitative RT-PCR

Strains were cultured for ~18 h as described in the colony sectoring assay section. Subsequently, colonies were resuspended in PBS, 4 mm glass beads were added, and the suspension subjected to vortex for 5 min to disperse aggregates. The resulting cell suspension was treated for 15 min at room temperature with RNAProtect reagent per the manufacturer’s instructions. Thereafter, RNA isolation, cDNA library preparation, and qPCR was performed as described previously (Mashruwala and Bassler, 2020).

#### Image acquisition

##### Time-lapse acquisition

Colonies were plated as described in the colony sectoring assay section. Thereafter, images of growing colonies were acquired using a Cytation 7 imaging plate reader (Biotek) using the attached temperature-controlled incubator at 30°C and a 43× air objective. Live-cell distribution was monitored using intensity from a chromosomally-integrated fluorescent reporter that constitutively produced the mKO protein (ex: 500 nm). Dead-cell distribution was monitored using staining intensity from SytoX (ex: 556 nm). The focal plane was maintained using the laser autofocus method. For each time point and in each acquisition channel, a 3×3 *xy*-montage of the colony was obtained and stitched together using the linear blend algorithm to form a single image. In every case, a depth of between 225 and 500 mm was sectioned. Maximum intensity *z*-projections were generated for each time point using the Biotek Gen5 software.

##### Bright-field and fluorescent stereo-microscope images

Colonies were plated as described above in the colony sectoring assay section. Following 2–3 days of growth, images were acquired using a Leica M125 stereo-microscope with a Leica MC170 HD camera.

#### Image analyses and quantitation

##### Time-projections

Projections of time-lapse data were obtained using customized Fiji scripts that performed the following sequence of events: First, image background subtraction was performed using a rolling ball radius of 1,000 pixels. Second, to account for shifts during imaging, the sequence of images was registered using the MultiStackReg Fiji plugin and the Rigid Body algorithm. Next, the registered image sequences were collapsed using maximum intensity projections. Ratio images were obtained using the Fiji Image calculator tool to divide pixel intensities across the entire image of the dead-cell channel by that for the live-cell channel. The grayscale time-projections and ratio images were pseudo-colored using Red (live channel), Cyan (dead channel) or Mpl-inferno (ratio image) look-up tables. To aid in visualization, the time-projection images were cropped at the colony boundaries and pixel intensities outside the colony boundaries were set to zero.

##### Weka machine learning-dependent image segmentation

Time-projection images for the first 38 h of colony growth were segmented and analyzed using the Fiji Trainable Weka Segmentation tool. First, a classifier model was trained to discriminate between the sectored and non-sectored regions of the colonies. The training dataset consisted of 25 time-projection images from the dead-cell channel using images of both parent and mutant colonies from experiments performed on multiple days. Using a custom Fiji script, the resulting classifier model was applied to time-projection images to obtain probability images in which each pixel in an image was assigned a probability of belonging to a particular image class. Regions of interest (ROI) were extracted from these probability images by thresholding using the RenyiEntropy algorithm followed by application of a combination of the filter and the particle size cutoff tools which were customized for each segmentation class. The obtained ROIs were manually curated for mis-segmentation, and the curated ROIs were used in measurements of area or intensities from the time-projection or the ratio images.

##### Space-time kymographs

Fluorescence time-course images of colony growth were analyzed using a custom MATLAB script. First, the center of the colony was located with an iterative centroid-finding algorithm using the fluorescent channel that monitored live cells, beginning at the first image acquisition at 8 h. To eliminate occasional sudden shifts due to mechanical noise, the sequence of images was registered in the *x*,*y* plane without rotation correction. Next, spatiotemporal fluorescence intensities in both the live- and dead-cell channels were extracted for kymograph analyses as follows: A region of interest consisting of a radial section, akin to a pie slice, was specified and manually verified to lack sectoring. Colony boundaries were determined using a fixed intensity threshold for the maximum fluorescence signals. Pixel intensities from both the live- and dead-cell channels were averaged in the circumferential direction within the radial section to obtain the averaged fluorescence intensity profile along the colony radius and over time. The obtained intensity values were used to construct kymograph profiles quantifying the space-time development of live and dead cells within the colony.

#### QUANTIFICATION AND STATISTICAL ANALYSIS

Sample variances were calculated using Excel. Statistical significance was calculated using a two-tailed Student’s *t* test appropriate for the sample variance.

## Supplementary Material

MMC1

MMC2

3

## Figures and Tables

**Table:** KEY RESOURCES TABLE

REAGENT or RESOURCE	SOURCE	IDENTIFIER
Bacterial and virus strains		
*Vibrio cholerae* wildtype C6706; Strep^R^	Bassler Lab Collection	BB-VC 90
*Vibrio cholerae* wildtype V52	Mekalanos Lab	AAM-18
*Vibrio cholerae* wildtype 2740–80; Strep^R^	Mekalanos Lab	AAM-20
2740–80; *luxO* A97E; variant	This work	AAM-1122
2740–80; *luxO* V120G; variant	This work	AAM-1123
2740–80; *lux O* 12aa deletion; variant	This work	AAM-1124
2740–80; *hapR* A52T; variant	This work	AAM-1125
2740–80; *hapR* R120P; variant	This work	AAM-1126
2740–80; *hapR* 2aa insertion; variant	This work	AAM-1127
2740–80; *hapR* IS200/IS605-like element insertion; variant	This work	AAM-1128
2740–80; *pyrG* T37I; variant	This work	AAM-1129
2740–80; *lacZ*::P*tac-mKO*	This work	AAM-890
2740–80; *lacZ*::P*tac-mKO* ∆*hapR*	This work	AAM-913
2740–80; *lacZ*::P*tac-mKO luxO* A97E	This work	AAM-933
2740–80; *lacZ*::P*tac-mKO* ∆*vasK*	This work	AAM-1130
2740–80; *lacZ*::P*tac-mKO* ∆*vpsL*	This work	AAM-907
2740–80; *lacZ*::P*tac-mKO* ∆*vgrG3-tsiV3*	This work	AAM-1006
2740–80; *lacZ*::P*tac-mKO* ∆*tseL-tsiV1*	This work	AAM-1016
2740–80; *lacZ*::P*tac-mKO* ∆*vasX-tsiV2*	This work	AAM-1025
2740–80; *lacZ*::P*tac-mKO* ∆*tseH-tsiH*	This work	AAM-1058
2740–80; *lacZ*::P*tac-mKO* ∆*vgrG3-tsiV3* ∆*vasX-tsiV2*	This work	AAM-1009
2740–80; *lacZ*::P*tac-mKO* ∆*tseL-tsiV1* ∆*tseH-tsiH*	This work	AAM-1059
2740–80; *lacZ*::P*tac-mKO* ∆*vasX-tsiV2* ∆*tseL-tsiV1* ∆*tseH-tsiH* (Only VgrG3-TsiV3^+^)	This work	AAM-1080
2740–80; *lacZ*::P*tac-mKO* ∆*vgrG3-tsiV3* ∆*tseL-tsiV1* ∆*tseH-tsiH* (Only VasX-TsiV2^+^)	This work	AAM-1083
2740–80; *lacZ*::Ptac-mKO ∆*vgrG3-tsiV3* ∆*vasX-tsiV2* ∆*tseH-tsiH* (Only TseL-TsiV1^+^)	This work	AAM-1068
2740–80; *lacZ*::P*tac-mKO* ∆*vgrG3-tsiV3* ∆*vasX-tsiV2* ∆*tseL-tsiV1* (Only TseH-TsiH^+^)	This work	AAM-1069
2740–80; *lacZ*::P*tac-mKO* ∆*vgrG3-tsiV3* ∆*vasX-tsiV2 ∆tseL-tsiV1* ∆*tseH-tsiH* (∆8)	This work	AAM-1028
2740–80; *lacZ*::P*tac-mKO* ∆*vasK* ∆*vgrG3-tsiV3* ∆*vasX-tsiV2* ∆*tseL-tsiV1* ∆*tseH-tsiH* (∆9)	This work	AAM-1027
2740–80; *lacZ*::P*tac-mKO* ∆*vgrG3-tsiV3* ∆*vasX-tsiV2* ∆*tseL-tsiV1* ∆*tseH-tsiH luxO* A97E (∆8 *luxO* A97E)	This work	AAM-1065
2740–80; *lacZ*::P*tac-mKO* ∆*vgrG3-tsiV3* ∆*vasX-tsiV2* ∆*tseL-tsiV1* ∆*tseH-tsiH* ∆*vasK luxO* A97E (∆9 *luxO* A97E)	This work	AAM-1131
2740–80; *lacZ*::P*tac-mKO* ∆*vgrG3-tsiV3* ∆*vasX-tsiV2* ∆*tseL-tsiV1* ∆*tseH-tsiH* ∆*vasK* ∆*vpsL* (∆9 ∆*vpsL*)	This work	AAM-1132
2740–80; *lacZ*::P*tac-mKO* ∆*vgrG3-tsiV3* ∆*vasX-tsiV2* ∆*tseL-tsiV1* ∆*tseH-tsiH* ∆*vasK luxO* A97E ∆*vpsL* (∆9 *luxO* A97E ∆*vpsL*)	This work	AAM-1133
2740–80; *lacZ*::P*tac-mKO VC1807*::P*tac-luxCDABE-*Spec^*R*^	This work	AAM-1134
2740–80; *lacZ*::P*tac-mKO* ∆*vasK* ∆*vgrG3-tsiV3* ∆*vasX-tsiV2* ∆*tseL-tsiV1* ∆*tseH-tsiH VC1807*::P*tac-luxCDABE-*Spec^*R*^ (∆9 T6SS-inactive prey)	This work	AAM-1135
2740–80; *lacZ*::P*tac-mKO* ∆*vgrG3-tsiV3* ∆*vasX-tsiV2* ∆*tseL-tsiV1* ∆*tseH-tsiH* ∆*vasK luxO* A97E *VC1807*:: P*tac-luxCDABE-*Spec^*R*^ (∆9 *luxO* A97E T6SS-inactive prey)	This work	AAM-1136
2740–80; *lacZ*::P*tac-mKO* ∆*vgrG3-tsiV3* ∆*vasX-tsiV2* ∆*tseL-tsiV1* ∆*tseH-tsiH* ∆*vasK* ∆*vpsL VC1807*:: P*tac-luxCDABE-*Spec^*R*^ (∆9 ∆*vpsL* T6SS-inactive prey)	This work	AAM-1137
2740–80; *lacZ*::P*tac-m YF2KO* ∆*vgrG3-tsiV3* ∆*vasX-tsiV2* ∆*tseL-tsiV1* ∆*tseH-tsiH* ∆*vasK luxO* A97E ∆*vpsL VC1807*::P*tac-luxCDABE-*Spec^*R*^ (∆9 *luxO* A97E ∆*vpsL* T6SS-inactive prey)	This work	AAM-1138
2740–80; *lacZ*::P*tac-mKO VC1807*:: P*luxC*-*mNeonGreen*-Spec^*R*^	This work	AAM-1139
2740–80; *lacZ*::P*tac-mKO* ∆*hapR VC1807*::P*luxC*-*mNeonGreen*-Spec^*R*^	This work	AAM-1140
C6706; *lacZ*::*luxC-lacZ VC1807*:: P*tac*-*mScarlet*-Spec^*R*^	This work	AAM-941
C6706; *lacZ*::*Ptac-mNeonGreen VC1807*::P*luxC*-*mScarlet*-Spec^*R*^	This work	AAM-857
*Saccharomyces cerevisiae*;YF2	Belden Lab Collection	AAM-25
*Escherichia coli*; Top10	Bassler Lab Collection	AAM-421
Recombinant DNA		
pRE112 (Purpose: Suicide vector with *sacB* counterselection)	Bassler Lab Collection	N/A
pRE112- *vgrG3-tsiV3* (Purpose: Chromosomal deletion of indicated genes)	This work	N/A
pRE112- *tseL-tsiV1* (Purpose: Chromosomal deletion of indicated genes)	This work	N/A
pRE112- *vasX-tsiV2* (Purpose: Chromosomal deletion of indicated genes)	This work	N/A
pRE112- *tseH-tsiH* (Purpose: Chromosomal deletion of indicated genes)	This work	N/A
pRE112- *vasK* (Purpose: Chromosomal deletion of indicated genes)	This work	N/A
pRE112- *luxO* A97E (Purpose: Chromosomal deletion of indicated genes)	This work	N/A
pRE112-*VC1807*::P*tac-luxCDABE-*Spec^R^ (Purpose: Chromosomal allele replacement at VC1807)	This work	N/A
pRE112-*VC1807*::P*luxC-mNeonGreen-*Spec^R^ (Purpose: Chromosomal allele replacement at VC1807)	This work	N/A
pKAS32 (Purpose: Suicide vector with Sm counterselection)	Bassler Lab Collection	N/A
pKAS32-*vpsL* (Purpose: Chromosomal deletion of indicated gene)	This work	N/A
pKAS32-*lacZ*::*mKO* (Purpose: Chromosomal allele replacement at *lacZ*)	This work	N/A
pEVS-pBAD (Purpose: Arabinose inducible gene expression vector)	This work	N/A
pEVS-pBAD-*tfoX*-*sacB*-pBAD-*qstR* (Purpose: *t6ss* overexpression plasmid (P*t6ss*-ON))	This work	N/A
P*vpsL-luxCDABE* (Purpose: Luciferase-based *vps* transcriptional reporter)	Bassler Lab Collection	N/A
P*luxC-luxCDABE* (Purpose: Luciferase-based transcriptional reporter of HapR activity)	Bassler Lab Collection	N/A
P*qrr*4-*luxCDABE* (Purpose: Luciferase-based transcriptional reporter of LuxO activity)	Bassler Lab Collection	N/A
Software and algorithms		
Original code (Image analyses and data visualization)	This work	Zenodo: https://doi.org/10.5281/zenodo.7076168
MATLAB (Image analyses and data visualization)	Mathworks	https://www.mathworks.com/products/matlab.html
Fiji (Image analyses and data visualization)	Schindelin et al., (2012)	https://ImageJ.nih.gov/ij/
Sigmaplot (Data visualization)	Inpixon	https://systatsoftware.com

## Highlights

- Quorum sensing and Type VI secretion drive cell death in *Vibrio cholerae* colonies
- Distinct T6SS toxins drive two spatiotemporal phases of cell death
- The first cell death phase occurs by kin-killing along the colony rim
- Kin-killing drives the emergence of variants fostering genetic diversity
