# Supplementary material for: Quorum-sensing- and type VI secretion-mediated spatiotemporal cell death drives genetic diversity in Vibrio cholera
Source: Cell. Author manuscript; Available in PMC 2022 Nov 1. (PMC9623500; doi:10.1016/j.cell.2022.09.003)
Supplement: MMC1 [file NIHMS1836874-supplement-MMC1.pdf]

Cell, Volume *185*

Supplemental information

Quorum-sensing- and type VI secretion-mediated spatiotemporal cell death drives genetic diversity in *Vibrio cholerae*

Ameya A. Mashruwala, Boyang Qin, and Bonnie L. Bassler

Supplemental Table 1, Related to Figure 2: Genotypes of isolated variants

**Table S1. Genotypes of isolated variants**		
**Number**	**Genotype**	**Function conferred**
1	*luxO* A97E	Gain-of-function
2	*luxO* V120G	Gain-of-function
3	*luxO* 12aa deletion (between 94-106aa)	Gain-of-function
4	*hapR* A52T	Attenuation-of-function
5	*hapR* R123P	Attenuation-of-function
6	*hapR* 2aa insertion (after 54th aa)	Loss-of-function
7	*hapR* ORF interrupted by IS200/IS605-like element	Loss-of-function
8	*pyrG* T37I (CTP synthase)	Not determined
9	3’ UTR region of *cspA* (cold-shock protein; region is known to influence *cspA* translation)	Not determined
