# Supplementary material for: Quorum-sensing- and type VI secretion-mediated spatiotemporal cell death drives genetic diversity in Vibrio cholera
Source: Cell. Author manuscript; Available in PMC 2022 Nov 1. (PMC9623500; doi:10.1016/j.cell.2022.09.003)
Supplement: 3 [file NIHMS1836874-supplement-3.pdf]

Supplemental figures

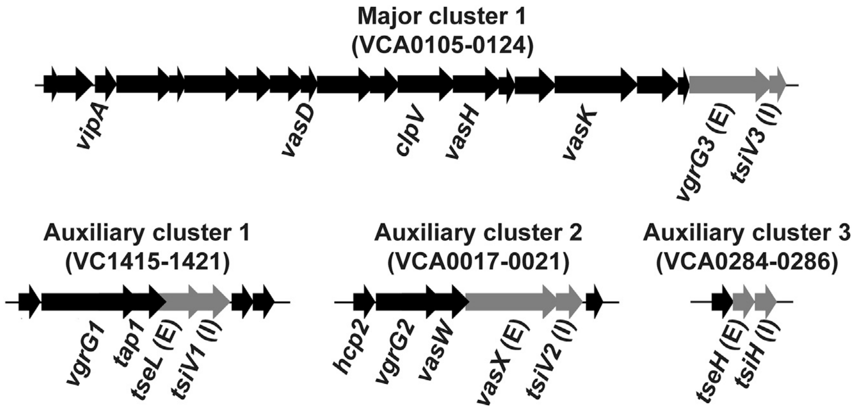

Figure S1. Arrangement of *V. cholerae t6ss* genes in four clusters, related to Figure 1
Select gene names are provided. Genes encoding effector and immunity proteins are depicted in gray and designated with, respectively, an E or I in parentheses (adapted from Metzger et al., 2016). The large cluster is on the major chromosome and the three auxiliary clusters are on the minor chromosome. The large cluster encodes the proteins that make the type VI secretion complex and one effector-immunity protein pair. Each of the auxiliary clusters encodes one effector-immunity protein pair.

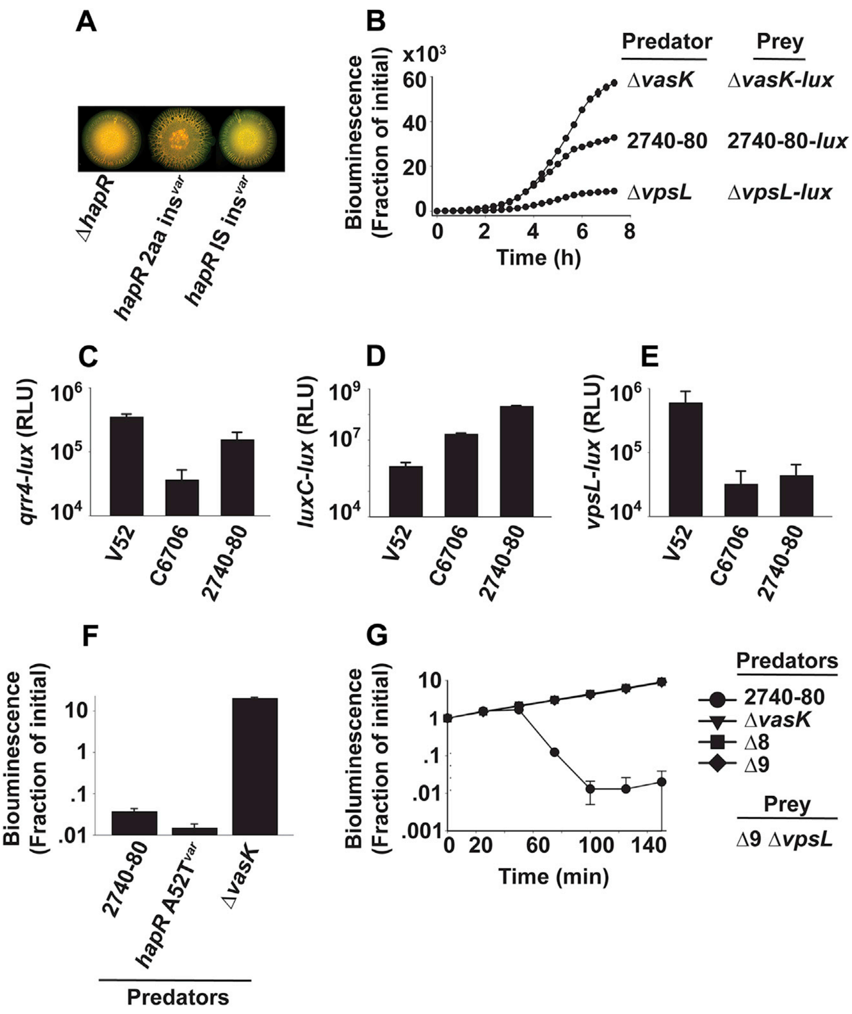

Figure S2. Colony morphologies, gene expression patterns, and T6SS killing activities for *V. cholerae* strains, related to Figures 2 and 6

(A) Brightfield stereo-microscope images of the morphologies of 2-day-old colonies of the *V. cholerae* 2740-80 Δ*hapR* strain and loss-of-function *hapR* variants. (B) Time-dependent survival of the indicated prey strains following challenge with the indicated *V. cholerae* predators. Error bars are present but may be obscured by the symbols (C–E) Light production from (C) *qrr*4-*lux*, (D) *luxC*-*lux*, and (E) *vpsL*-*lux* in the indicated strains. Relative light units (RLU) are defined as bioluminescence output divided by culture optical density. (F) Inter-bacterial T6SS killing assay measuring survival of T6SS-inactive *E. coli* prey following challenge with the indicated *V. cholerae* predators. (G) Inter-bacterial T6SS killing assay measuring time-dependent survival of *V. cholerae* 2740-80 Δ9 Δ*vpsL* prey cells following challenge with the indicated *V. cholerae* predators. In Panels B, F and G, the prey strains constitutively express luciferase. Thus, bioluminescence output is a proxy for live prey cells. Data represent the average values from four (C–E) or three (B,F,G) biological replicates and error bars show SDs.

CellPress
OPEN ACCESS

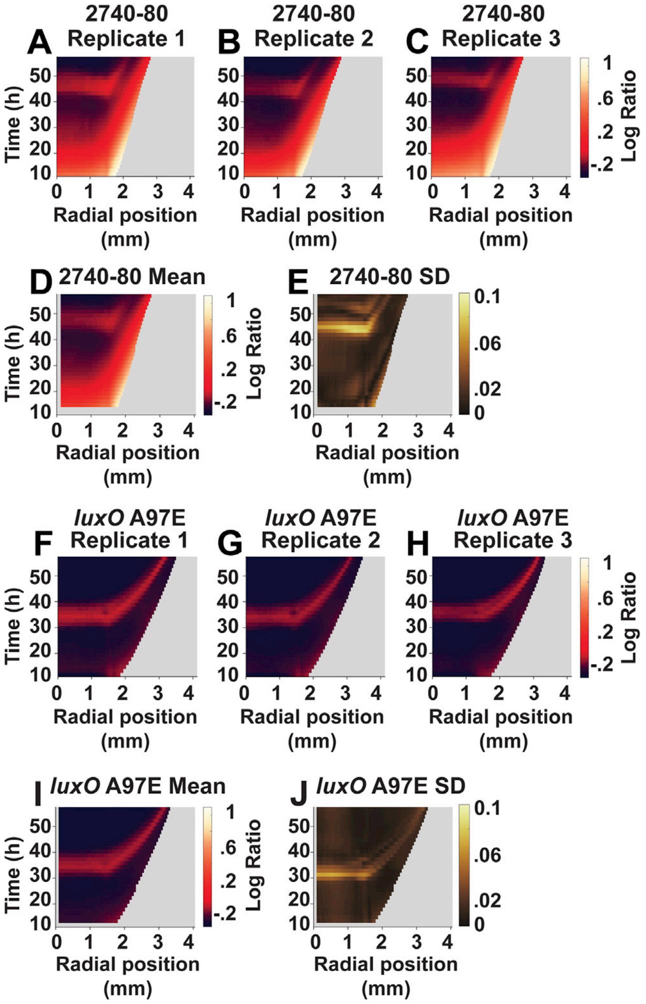

Figure S3. Minimal variation occurs in cell death patterns between colonies of the same genotype, related to Figures 3, 4, and 6

(A-C, F–H) Logarithmic ratio kymographs for three colonies of the indicated strains are shown. (D,I) Mean logarithmic ratio kymographs for data in panels A-C (Panel D) and panels F–H (Panel I). (E,J) Standard deviations of the kymographs in panels A-C (Panel E) and panels F–H (Panel J). The mean and SD were computed following registration in space along the biofilm radius and shifting in time to account for slight variations in growth. The SD between colonies is <3% for Phase 1 cell death and <10% for Phase 2 cell death. Because Phase 2 cell death propagates in two directions, higher colony-to-colony variation is anticipated. Kymograph data treated as described for Figure 4.

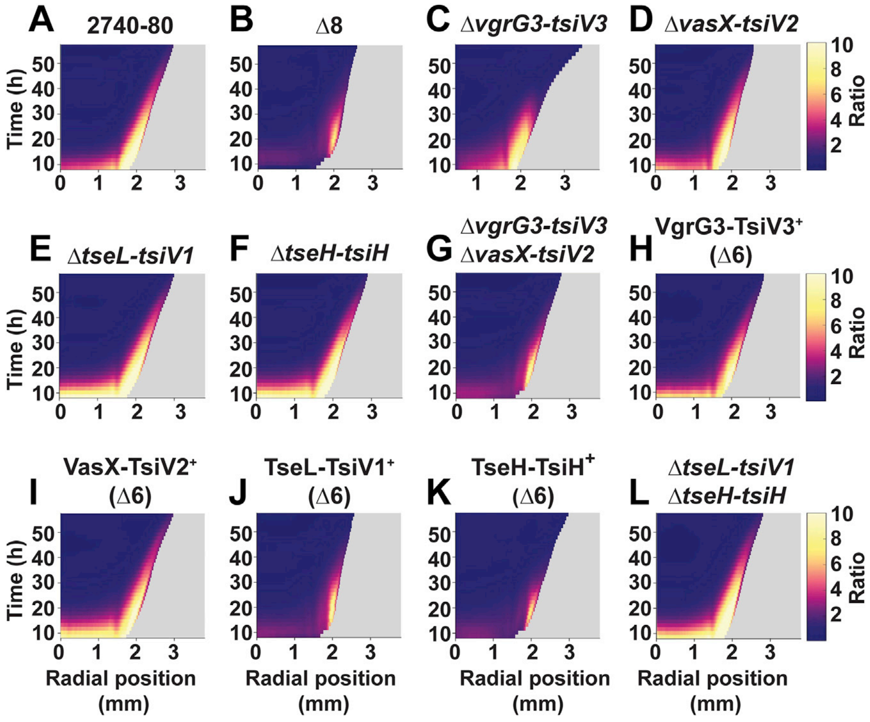

Figure S4. The VgrG3-TsiV3 and VasX-TsiV2 T6SS effector-immunity pairs drive Phase 1 cell death in *V. cholerae* 2740-80, related to Figure 5
Linear ratio kymographs for the indicated strains. The linear scale emphasizes differences in Phase 1 cell death. Kymograph data treated as described for Figure 4. Kymographs from one colony are representative of results from 3 to 9 colonies for each strain. The Phase 1 cell death shown in panel C for the Δ*vgrG3-tsiV3* strain appears to end abruptly. This feature is due to a technical limitation. Specifically, the Δ*vgrG3-tsiV3* strain displays hyper-sectoring (see Figure S6). Thus, by late Phase 1 (~34–40 h), the outer regions of Δ*vgrG3-tsiV3* colonies are composed almost entirely of sectors. Because we exclude sectored regions from the kymograph analyses (see STAR Methods), Phase 1 appears abbreviated in this strain, but that is not the case.

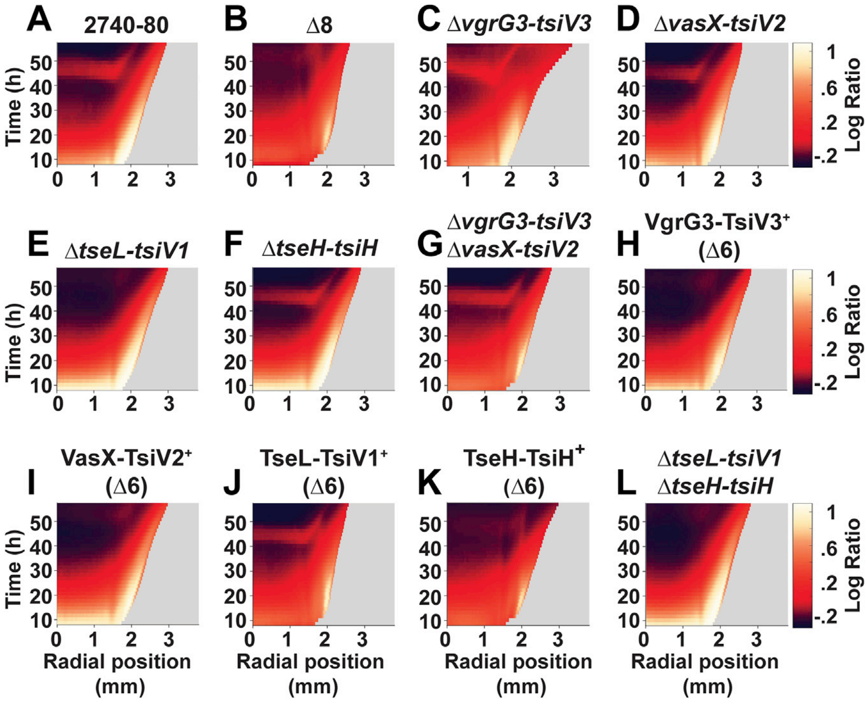

Figure S5. The TseH-TsiH effector-immunity pair is dispensable for T6SS-mediated cell death in *V. cholerae* 2740-80, related to Figure 5
Logarithmic ratio kymographs for the indicated strains. Kymograph data treated as described for Figure 4. Kymographs from one colony are representative of results from 2 to 6 colonies for each strain. The abrupt truncation of Phase 1 cell death shown in panel C for the Δ*vgrG3-tsiV3* strain is a consequence of the imaging limitation in which sectors are omitted and is also described in the Figure S4 legend. The order in which the strains are arranged is identical to that in Figure S4.

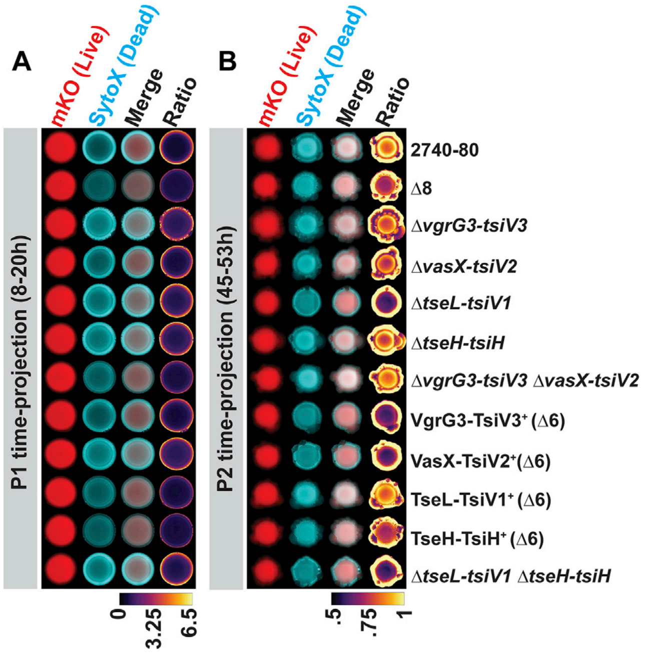

Figure S6. The VgrG3-TsiV3 and VasX-TsiV2 effector-immunity pairs drive Phase 1 cell death, while the TseL-TsiV1 effector-immunity pair drives Phase 2 cell death in *V. cholerae* 2740-80, related to Figure 5

Time-projections show cell death and sectoring for the indicated phases and strains. The order in which the strains are arranged is identical to that in Figures S4 and S5.

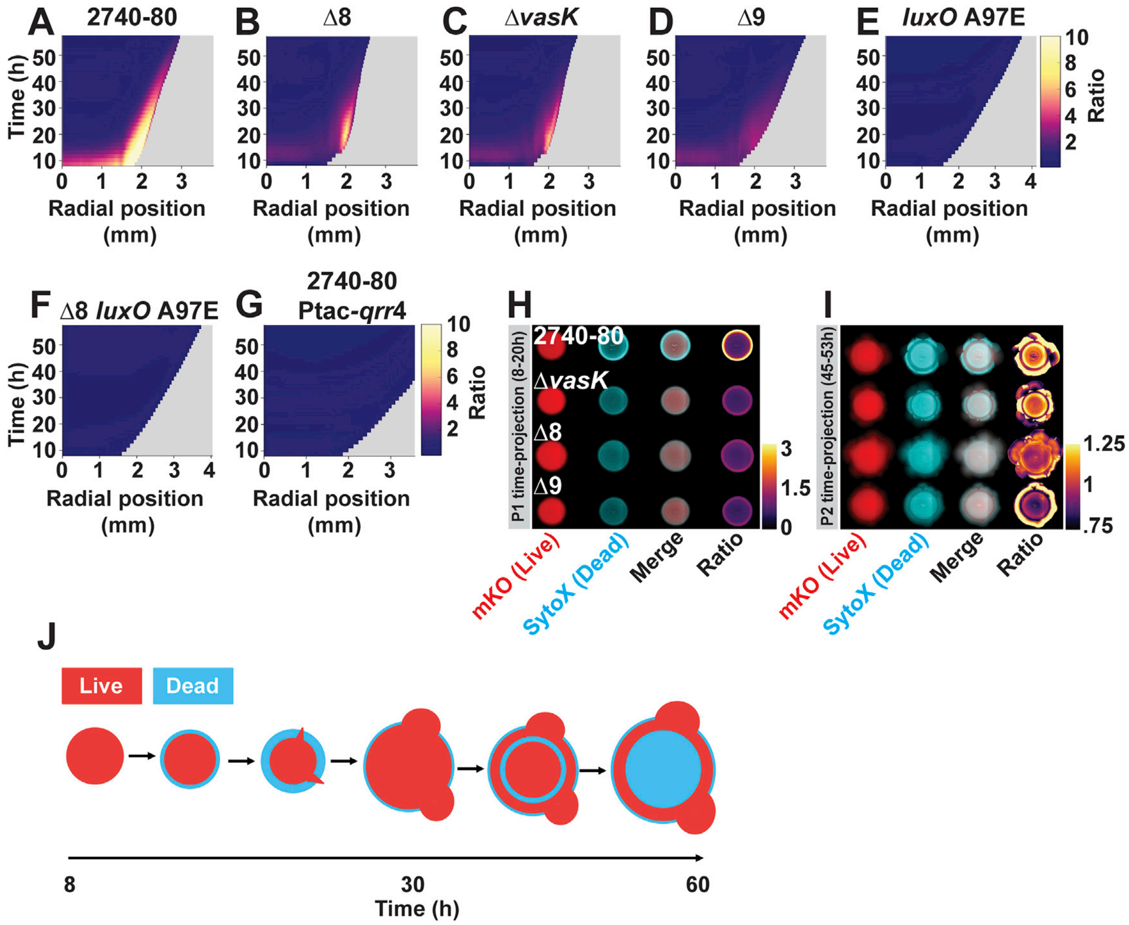

Figure S7. Cell death patterns for *V. cholerae* 2740-80 strains and a model for how spatiotemporal cell death drives the formation of colony sectors, related to Figures 5 and 6, and Discussion

(A–G) Strains lacking *vasK* or harboring *luxO* A97E are deficient in Phase 1 cell death. Linear ratio kymographs for the indicated strains. Kymograph data treated as described for Figure 4. Kymographs from one colony are representative of results from 3 to 9 colonies for each strain. (H and I) *V. cholerae* 2740-80 lacking all T6SS components displays less sectoring than *V. cholerae* 2740-80 and the VasK-dependent T6SS-injection machinery is required for Phase 1 cell death but is dispensable for Phase 2 cell death. Time-projections showing cell death and sectoring for the indicated strains and phases. (J) A model for cell death and sectoring in *V. cholerae* 2740-80 colonies. Colonies of *V. cholerae* strain 2740-80 undergo two-phase T6SS-mediated spatiotemporal cell death. Phase 1 initiates at ~8 h post inoculation, occurs along the colony periphery, is driven by the VgrG3 and VasX toxins and is regulated by QS. At ~18 h post inoculation, sectors emerge along the colony rim, i.e., from regions displaying high Phase 1 cell death. Cells in sectors undergo low cell death. Sectoring does not occur in a strain that is locked into the low cell density QS mode and that does not undergo Phase 1 cell death. Thus, Phase 1 cell death is key for sectoring to occur and, therefore, for enhanced genetic diversity to arise in the population. At ~42 h, Phase 2 cell death initiates as a ring in the colony interior. Phase 2 cell death is driven by the TseL toxin and does not require the T6SS injection apparatus. Cell death propagates inward and outward from the initial ring.

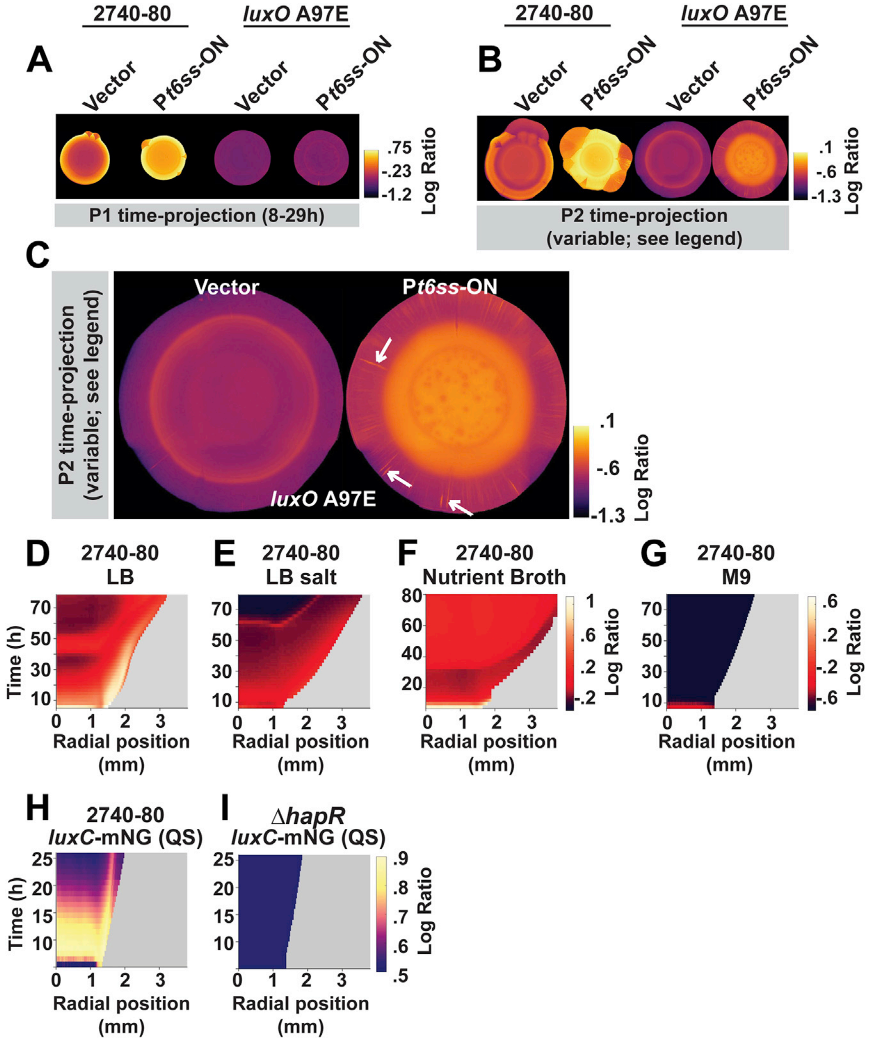

Figure S8. Cell death and colony sectoring phenotypes in *V. cholerae* strains, related to Figure 7 and Discussion

(A-B) Logarithmic ratio time-projections showing cell death and sectoring for the indicated phases in strains carrying either an empty vector (denoted: Vector) or the vector carrying P*t6ss*-ON. Phase 2 projection timing as in Figure 6I. (C) The *V. cholerae* 2740-80 *luxO* A97E strain carrying P*t6ss*-ON forms radial sectors. Shown are enlarged time-projection images of colonies of the indicated strains from Panel B. The white arrows point to radial sectors. Such sectors are not apparent in the strain carrying the empty vector. These data accompany Figure 7. (D–G) Phase 1 cell death along the colony rim is suppressed in *V. cholerae* 2740-80 grown on rich medium or when provided glucose. Logarithmic ratio kymographs for *V. cholerae* 2740-80 grown on LB (D), LB medium supplemented with oceanic levels of NaCl (E), nutrient broth (F), and M9 defined medium with glucose (G) (H–I) QS activity in *V. cholerae* 2740-80 is higher along the colony rim than in the center during Phase 1 cell death. Logarithmic ratio kymographs for QS activity from the indicated *V. cholerae* 2740-80 strains constitutively producing mKO to mark live cells and that also encode a QS-activated reporter (*luxC*-mNG). The time required for maturation of mKO and mNG differs, a feature that could influence the normalized data. Kymograph data in D–G treated as described for Figure 4. Kymographs from one colony are representative of results from 2 to 4 colonies for each condition. In D–G, Phase 1 cell death did not occur when cells were grown on nutrient broth or M9 medium. Phase 2 cell death took place in nutrient broth, albeit with altered timing and spatial distribution compared to growth on LB medium. Phase 2 cell death did not occur in M9 medium. Addition of NaCl muted both the Phase 1 and Phase 2 cell death phenotypes.
